# Evolutionary history of human colitis-associated colorectal cancer

**DOI:** 10.1136/gutjnl-2018-316191

**Published:** 2018-07-10

**Authors:** Ann-Marie Baker, William Cross, Kit Curtius, Ibrahim Al Bakir, Chang-Ho Ryan Choi, Hayley Louise Davis, Daniel Temko, Sujata Biswas, Pierre Martinez, Marc J Williams, James O Lindsay, Roger Feakins, Roser Vega, Stephen J Hayes, Ian P M Tomlinson, Stuart A C McDonald, Morgan Moorghen, Andrew Silver, James E East, Nicholas A Wright, Lai Mun Wang, Manuel Rodriguez-Justo, Marnix Jansen, Ailsa L Hart, Simon J Leedham, Trevor A Graham

**Affiliations:** 1 Barts Cancer Institute, Barts and The London School of Medicine and Dentistry, Queen Mary University of London, London, UK; 2 Inflammatory Bowel Disease Unit, St Mark’s Hospital, London, UK; 3 Wellcome Trust Centre for Human Genetics, University of Oxford, Oxford, UK; 4 Department of Computer Science, University College London, London, UK; 5 Centre for Mathematics and Physics in the Life Sciences and Experimental Biology (CoMPLEX), University College London, London, UK; 6 Department of Cell and Developmental Biology, University College London, London, UK; 7 Blizard Institute, Barts and The London School of Medicine and Dentistry, Queen Mary University of London, London, UK; 8 Department of Histopathology, The Royal London Hospital, London, UK; 9 Department of Gastroenterology, University College London Hospital, London, UK; 10 Department of Histopathology, Salford Royal NHS Foundation Trust, University of Manchester, Manchester, UK; 11 Cancer Genetics and Evolution Laboratory, Institute of Cancer and Genomic Sciences, University of Birmingham, Birmingham, UK; 12 Translational Gastroenterology Unit, Nuffield Department of Medicine, University of Oxford, John Radcliffe Hospital, Oxford, UK; 13 Cellular Pathology, John Radcliffe Hospital, Oxford, UK; 14 Department of Histopathology, University College London Hospital, London, UK

**Keywords:** inflammatory bowel disease, colorectal cancer, dysplasia, IBD - genetics

## Abstract

**Objective:**

IBD confers an increased lifetime risk of developing colorectal cancer (CRC), and colitis-associated CRC (CA-CRC) is molecularly distinct from sporadic CRC (S-CRC). Here we have dissected the evolutionary history of CA-CRC using multiregion sequencing.

**Design:**

Exome sequencing was performed on fresh-frozen multiple regions of carcinoma, adjacent non-cancerous mucosa and blood from 12 patients with CA-CRC (n=55 exomes), and key variants were validated with orthogonal methods. Genome-wide copy number profiling was performed using single nucleotide polymorphism arrays and low-pass whole genome sequencing on archival non-dysplastic mucosa (n=9), low-grade dysplasia (LGD; n=30), high-grade dysplasia (HGD; n=13), mixed LGD/HGD (n=7) and CA-CRC (n=19). Phylogenetic trees were reconstructed, and evolutionary analysis used to reveal the temporal sequence of events leading to CA-CRC.

**Results:**

10/12 tumours were microsatellite stable with a median mutation burden of 3.0 single nucleotide alterations (SNA) per Mb, ~20% higher than S-CRC (2.5 SNAs/Mb), and consistent with elevated ageing-associated mutational processes. Non-dysplastic mucosa had considerable mutation burden (median 47 SNAs), including mutations shared with the neighbouring CA-CRC, indicating a precancer mutational field. CA-CRCs were often near triploid (40%) or near tetraploid (20%) and phylogenetic analysis revealed that copy number alterations (CNAs) began to accrue in non-dysplastic bowel, but the LGD/HGD transition often involved a punctuated ‘catastrophic’ CNA increase.

**Conclusions:**

Evolutionary genomic analysis revealed precancer clones bearing extensive SNAs and CNAs, with progression to cancer involving a dramatic accrual of CNAs at HGD. Detection of the cancerised field is an encouraging prospect for surveillance, but punctuated evolution may limit the window for early detection.

**Significance of this study**

**What is already known on this subject?**

- IBD confers an increased lifetime risk of developing colorectal cancer (CRC).
- Colitis-associated CRC (CA-CRC) is molecularly distinct from sporadic CRC, for example, there is a higher frequency of *TP53* mutation while *APC* and *KRAS* mutations occur at lower frequency.
- Endoscopic surveillance for early detection of CA-CRC is fraught with challenges, and the rate of interval cancers remains very high.

**What are the new findings?**

- We provide the first quantification of the intratumour genetic heterogeneity in CA-CRC, and trace the spatiotemporal evolution of cancer from preneoplastic lesions and non-dysplastic mucosa, using multiregion exome sequencing of fresh-frozen samples.
- Evolutionary divergence of sporadic and colitis-associated cancers begins in the non-dysplastic colitic mucosa, well before the emergence of an identifiable lesion.
- Rapid ‘punctuated’ evolution of copy number alterations commonly demarcates the transition between low-grade and high-grade dysplasia.

**Significance of this study**

**How might it impact on clinical practice in the foreseeable future?**

- Knowledge of the early genetic events that distinguish sporadic and colitis-associated disease can be exploited for subsequent biomarker development to provide precision molecular diagnosis of true colitis-associated lesions versus incidental sporadic disease.
- We show that the burden of aneuploidy increases with lesion grade, suggesting that aneuploidy may be a useful biomarker to risk-stratify low grade lesions.
- We identify recurrent early genetic mutations in the development of CA-CRC; these are potentially useful as targets for cancer chemoprevention.

## Introduction

Patients with IBD have an increased risk of developing colorectal cancer (CRC) compared with the colitis-free population,^1 2^ and this risk is closely associated with the extent,^2 3^ duration^1^ and severity^4^ of inflammation. There are a number of key phenotypic features that differentiate colitis-associated CRC (CA-CRC) from the more common sporadic CRC (S-CRC): CA-CRCs occur more frequently in patients of younger age,^2 5 6^ they are more often synchronous^6^ and they have higher frequency of mucinous or signet ring cell histology.^7^ Furthermore, rather than developing from a polypoid adenoma, CA-CRC is thought to often arise from flat dysplasia with indistinct margins, in a field of concomitant inflammation, scarring and pseudopolyposis, making endoscopic detection and resection challenging.^8^

The efficacy of colonoscopic surveillance programmes in patients with IBD is poor compared with that of the conventional bowel screening programme. The rate of interval cancers in IBD is reported to be up to 30%, despite patients adhering to intensive surveillance protocols.^9^ Furthermore, the chance of identifying endoscopically undetected CA-CRC in patients undergoing immediate panproctocolectomy for dysplasia is approximately 25% for low-grade dysplasia (LGD) and 50% for high-grade dysplasia (HGD).^6^ These observations highlight an unmet clinical need for insight into the molecular events underpinning the development of CA-CRC, and the temporal patterns by which they accrue.

At the molecular level, the sequence of events leading to CA-CRC is distinct from S-CRC: most notably *TP53* mutation is typically an early event in the former, detected in precancerous neoplasms^10^ or even in non-neoplastic mucosa,^11 12^ whereas *TP53* mutations are rare in the adenomatous precursors of S-CRC.^13 14^ Aneuploidy can be present in non-dysplastic colitic epithelium,^15 16^ suggesting a role for chromosomal instability early in the genesis of CA-CRC. Furthermore, *APC* 17 and *KRAS* 18 mutations are reported to be less prevalent in CA-CRC than in S-CRC. Moreover, recent exome^19^ and targeted sequencing^20^ of CA-CRCs has revealed a distinct set of genes bearing single nucleotide alterations (SNA) in CA-CRCs. The temporal history of this unique mutational complement is undetermined, but it is likely to be a consequence of the different selective pressures in CA-CRC versus S-CRC that result from chronic exposure to the inflammatory environment of the colitic bowel.^19 20^ Further characterisation of the differences in evolutionary trajectory between CA-CRC and S-CRC will guide improvements in clinical detection, molecular biomarker risk stratification and cancer chemoprevention, and offer new opportunities for targeted therapies.^21^

Here we have used multiregion exome sequencing and genome-wide copy number (CN) analysis to generate phylogenetic trees and determine the evolutionary history of CA-CRC.

## Materials and methods

### Patient samples

Samples were obtained from University College and St Mark’s Hospitals, London, under multicentre ethical approval (London Stanmore committee, 11/LO/1613) and the UCLH Biobank, with patients giving informed consent for prospectively collected tissue. Additional samples were from the Manchester Cancer Research Centre Biobank (Project 13_NIWR_01), the Oxford University Hospitals (MREC 10/H0604/72) and the Royal London Hospital (REC 13/LO/1271). Clinical characteristics can be found in online supplementary tables 1–3. Neoplastic grading was performed by analysis of H&E stained sections by at least three expert histopathologists (MM, MRJ, MJ, LMW).

10.1136/gutjnl-2018-316191.supp1 Supplementary data

*Fresh-frozen samples:* Samples were collected from patients with long-standing (>9 years) ulcerative colitis (UC) or Crohn’s disease (CD) undergoing surgery to remove CA-CRC (ie, panproctocolectomy). For 10 patients we collected between two and five spatially distinct carcinoma samples, at least one sample of non-dysplastic mucosa and any synchronous lesions that were spatially separate from the primary carcinoma. For an additional three patients a single carcinoma sample was obtained. These samples were immediately snap-frozen in liquid nitrogen and stored at −80C.

*Formalin-fixed paraffin-embedded (FFPE) samples:* FFPE samples were obtained with ethical approval as stated above. Twelve CA-CRCs were obtained from the archive of St Mark’s Hospital, London, for single nucleotide polymorphism (SNP) array analysis. A further 81 regions from 39 lesions of mixed histology, representing 19 patients with UC (LGD: 38, mixed HGD/LGD: 12, HGD: 23, CA-CRC: 7, pseudopolyp: 1) were obtained for analysis by low-pass whole genome sequencing (LP-WGS). An additional 25 sporadic tubulovillous adenomas were obtained from Oxford for SNP array analysis. Thirteen S-CRCs were obtained from University College London Hospital for immunohistochemical analysis of β-catenin expression.

### Sequencing

Exome sequencing was performed on multiregion samples of the n=13 fresh-frozen cases described above (for details see online supplementary materials and methods). Select variants were validated with Sanger sequencing (online supplementary table 4).

10.1136/gutjnl-2018-316191.supp2 Supplementary data

### BaseScope in situ hybridisation

In situ hybridisation for the *KRAS* G12A mutation was performed as previously described^22^ using the BaseScope assay according to the manufacturer’s guidelines (Advanced Cell Diagnostics, Newark, CA). Stained slides were digitised and mutant regions (displaying punctate red signal) were manually annotated using Adobe Photoshop CS6.

### Data availability

Raw sequence data and SNP array calls, together with processed data, are available at the European Genome-Phenome Archive (EGA) with accession number EGAS00001003028.

### Statistical analysis

For testing two independent groups the Mann-Whitney U test was used. For testing more than two groups the Kruskal-Wallis test was used. Comparison of mutation frequency and arm-level copy number alteration (CNA) frequency between S-CRC and CA-CRC was performed using Fisher’s exact test. Results were considered significant when *q*<0.05, after using the Benjamini-Hochberg method to control the false discovery rate. For further details please see online supplementary materials and methods.

## Results

### Mutational burden in CA-CRCs and surrounding mucosa

We performed whole exome sequencing (WXS) on fresh-frozen specimens representing CRC arising on a background of long-standing (>9 years) colitis (n=10 UC, n=3 Crohn’s colitis). Three patients had concomitant primary sclerosing cholangitis (PSC). For 10 of these patients we generated multiregion exome data by sequencing regions of carcinoma and non-dysplastic mucosa, in addition to other neoplastic lesions where possible (see online supplementary table 1) and called SNAs and small insertions and deletions (indels) against matched normal DNA (11 cases were called against whole blood, one against microdissected muscle and one against microdissected lymphocytes; online supplementary tables 5 and 6). Data from one patient (UC10) did not pass our quality control criteria for SNA/indel identification, and was therefore excluded from further mutational assessment and used only for CNA analysis. SNAs in *TP53* (in four carcinomas) and *KRAS* (in two carcinomas) were validated using Sanger sequencing of microdissected carcinoma and non-dysplastic tissue (online supplementary figure 1A,B). Microsatellite instability (MSI) was identified in two cases (UC02 and UC09) from their high mutation burden (median 982 non-synonymous mutations, 33 SNAs/Mb, online supplementary table 5), and verified using MSI sensor software. UC09 harboured a *BRAF* V600E mutation, UC02 was confirmed as MSI using fragment analysis (online supplementary figure 2A) and had loss of PMS2 protein expression by immunohistochemistry (IHC, online supplementary figure 2B). Single nucleotide variant and indel calls are provided in online supplementary table 6.

10.1136/gutjnl-2018-316191.supp3 Supplementary data

10.1136/gutjnl-2018-316191.supp4 Supplementary data

**Figure 1:**
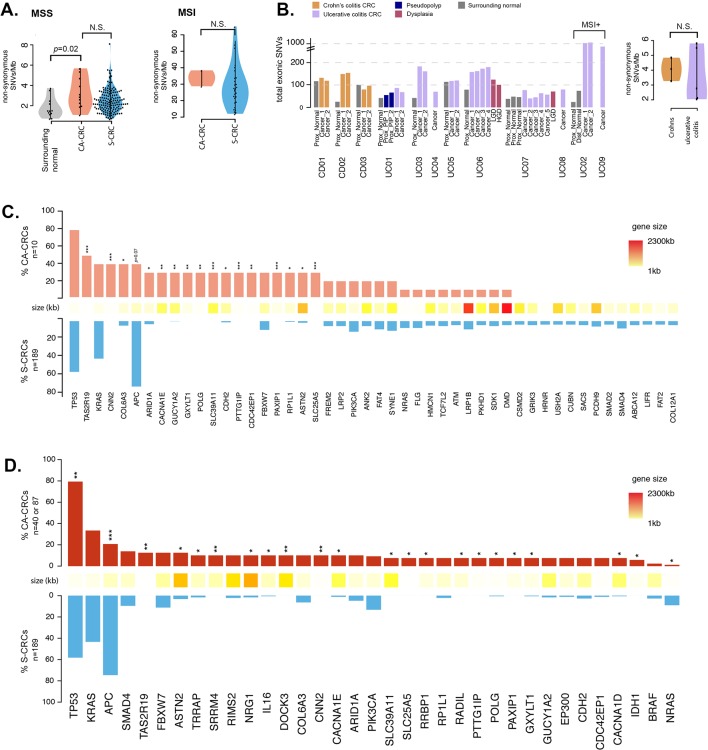
Analysis of single nucleotide alteration (SNA) burden in CA-CRC. (A) Analysis of mutation frequency in CA-CRC versus S-CRC (MSS=left panel, CA-CRC n=10, S-CRC n=191; MSI=right panel, CA-CRC n=2, S-CRC n=35). (B) Analysis of mutation burden and heterogeneity in UC versus Crohn’s colitis cases. Data are shown per biopsy (left panel) and per patient (right panel). (C) Frequency of CA-CRC (n=10) and S-CRC (n=191) MSS cases with non-synonymous SNAs in genes mutated in >20% of CA-CRCs or >5% of S-CRCs. *p<0.05; ***p<0.001 by Fisher’s exact test. (D) Meta-analysis displaying the frequency of recurrently mutated genes in CA-CRC (n=40 or n=87) and S-CRCs (n=189), incorporating data from the current study and two previous studies.^19 20^ *p<0.05; **p<0.01; ***p<0.001 by Fisher’s exact test. CA-CRC, colitis-associated CRC; CRC, colorectal cancer; MSI, microsatellite instability; MSS, microsatellite stable; NS, not significant by the Mann-Whitney test; S-CRC, sporadic CRC; SNV, single nucleotide variant.

There was a non-significant increase in SNA burden in CA-CRCs compared with S-CRC (The Cancer Genome Atlas (TCGA) data set^23^); microsatellite stable (MSS) CA-CRCs had a median of 104 (range 57–172) non-synonymous mutations per carcinoma (3.0 SNAs/Mb, range 1.2–5.8), 20% higher than TCGA S-CRCs^23^ (median 2.5 SNAs/Mb, figure 1A; p=0.1). MSI CA-CRCs showed the same suggestion of an ~20% increase in SNA burden compared with their S-CRC MSI counterparts (33 vs 27 SNAs/Mb, figure 1) although again this was not statistically significant (p=0.4).

We analysed mutational burden in non-dysplastic tissue by extracting DNA from a region of approximately 0.5 cm by 0.5 cm by laser capture microdissection (eight cases) or by needle macrodissection (two cases). Non-dysplastic tissue surrounding CA-CRCs showed considerable mutational burden (median 1.6 SNAs/Mb). On average, this was significantly lower than that of CA-CRCs themselves (p=0.02, figure 1A), but was comparable in three cases (cases CD01, CD03, UC05; figure 1B). These data suggest that the underlying inflammatory process exerts a mutational toll on the background mucosa, and implies that the SNA burden alone cannot necessarily differentiate untransformed and malignant cells.

### Recurrently mutated genes in CA-CRC

We searched for a novel mutational complement in our CA-CRCs by comparing the recurrently mutated genes with those in S-CRCs (TCGA data set^23^), and removing spurious mutation calls identified in previous studies.^24 25^ In figure 1C we report mutational frequency of 48 genes, 20 of which are recurrently mutated in ≥30% of CA-CRCs, and 33 of which are mutated in ≥5% of S-CRCs (five genes are mutated in both ≥30% of CA-CRCs and ≥5% of S-CRCs).

*TP53* was mutated in every MSS CA-CRC examined, with 8/10 carrying at least one non-synonymous exonic *TP53* mutation, and the remaining two cancers carrying splice site mutations (online supplementary table 7). A total of 12 exonic *TP53* mutations were identified (eight missense, two non-sense, two frameshift) and eight of these mutations (75%) were within the DNA-binding domain (amino acids 102–292).^26^ One carcinoma (UC01) even exhibited three different DNA-binding domain missense mutations, two of which have been reported to confer oncogenic gain of function to the p53 protein.^27^ Interrogation of the UC01 raw variant allele frequencies and estimated cancer cell fractions revealed that all three mutations are present at near-clonal frequency, thus it is probable that they arose at a similar time within the progenitor cell of the lesion. Furthermore, we were able to deduce that the p.Y234H and p.G245S mutations are present on the same allele (as they were present on the same raw reads). This case is an example of convergent evolution, indicating a strong selective pressure for loss of normal p53 function.

There was a prevalence of *TP53* mutations in exon 7 (5/12, 42%), with two of these occurring at codon 245, a known hotspot in S-CRC.^28^ It is noteworthy that in case UC07, the LGD biopsy displayed a *TP53* p.R306X mutation, yet this variant was absent in the carcinoma itself, where instead a *TP53* splice site mutation was detected. *TP53* mutation frequency in our cohort was not significantly higher than that of S-CRCs (CA-CRC=80% vs S-CRC=58%, *q*=0.4). Other common S-CRC driver genes that were mutated at similar frequency in our CA-CRC cohort included *KRAS* (CA-CRC=40% vs S-CRC=43%, *q*=1.0), *PIK3CA* (20% vs 13%, *q*=0.9) and *FBXW7* (30% vs 11%, *q*=0.2).

Confirming previous single-gene^17^ and next-generation sequencing (NGS)^19 20^ studies, we observed that mutation of the most common S-CRC driver gene *APC* was less prevalent in CA-CRC (CA-CRC=40%, S-CRC=75%, *q*=0.07). As previously noted,^19^ *APC* mutations were more prevalent in CD (2/3 cases, 67%) than UC (2/7 cases, 29%; p=0.5). One CA-CRC carried a mutation in β-catenin (exon 3, p.D32G). We compared the levels of nuclear and cytoplasmic β-catenin (measured by IHC) in CA-CRC (n=8) and S-CRC (n=13; online supplementary figure 3A,B), and found that cytoplasmic β-catenin is not significantly different (p=0.25 by Fisher’s test), however a greater proportion of CA-CRC samples have low levels of nuclear β-catenin (p=0.024 by Fisher’s test). CA-CRC samples with *APC* mutations did not have significantly higher levels of β-catenin (online supplementary figure 3A).

Sixteen genes were observed to be significantly more frequently mutated in CA-CRCs than in S-CRCs (figure 1C), therefore may be involved specifically in CA-CRC pathogenesis. Thirty-five per cent of the putative CA-CRC driver mutations were ‘stopgains’, ‘indels’ or SNAs that were annotated as ‘cancer’ by the FATHMM software,^29^ and the remaining 65% were of ‘unknown’ or ‘passenger’ status (see online supplementary table 6). Notably, the list of putative CA-CRC drivers includes the well-characterised S-CRC driver gene *ARID1A* (CA-CRC=30%, S-CRC=5%, *q*=0.048). Also of interest is the gene *CDH2* (N-cadherin), as the expression of mutant N-cadherin in mouse small intestine has been shown to induce a Crohn’s-like phenotype.^30^ Other notable genes that were mutated in at least 30% of CA-CRC, but mutated at very low frequency in S-CRC included *POLG* (DNA polymerase gamma, involved in mitochondrial DNA replication) and *PAXIP1* (involved in maintaining genome stability). Online supplementary table 8 provides a description of all 16 putative CA-CRC drivers and their functions.

We combined our CA-CRC mutational frequencies with previously published WXS^19^ (30 MSS CA-CRCs) and targeted sequencing^20^ (47 CA-CRCs) data sets (figure 1D and online supplementary table 9). With this larger sample size (n=87) we found significantly increased *TP53* mutation frequency in CA-CRC (79% vs 58%, *q*=0.005) and very significantly reduced *APC* mutation frequency (20% vs 75%, *q*<0.001). A total of 22 genes showed significantly different mutation frequency between CA-CRC and S-CRC, though there was little overlap between the set of mutant genes in the different studies (figure 1D and online supplementary table 9). Together, these data indicate a potentially novel set of SNA driver mutations in CA-CRC, over-and-above existing S-CRC driver genes.

### Evolutionary history of SNAs

**Figure 2:**
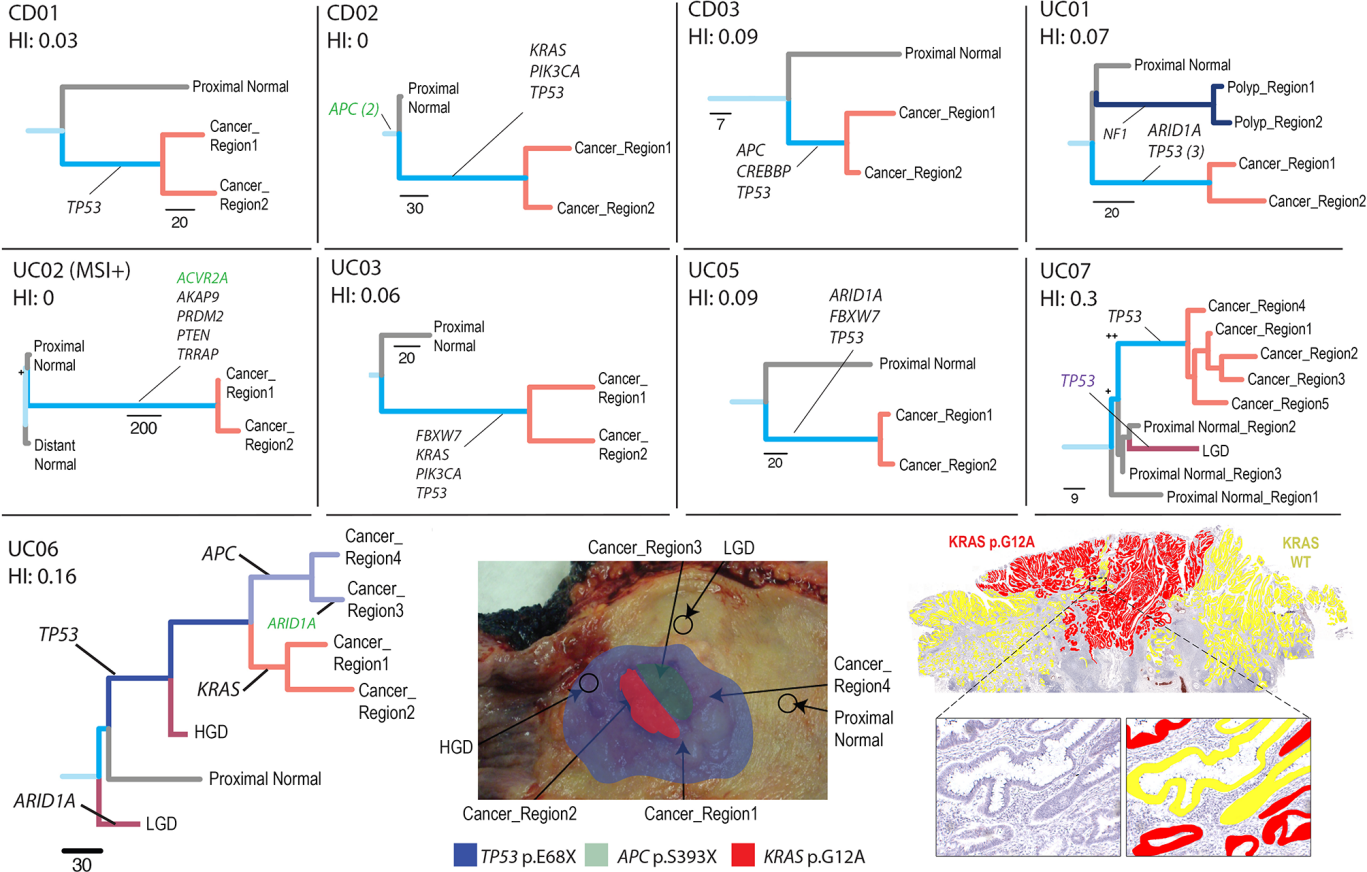
Analysis of single nucleotide alteration (SNA) phylogeny in colitis-associated CRC (CA-CRC). Phylogenetic trees were produced using maximum parsimony and multiregion whole exome sequencing (WXS) of CA-CRCs. Branches are labelled with SNA drivers (black), indel drivers (green) and splice site variants (purple). The lower panel shows case UC06 in further detail, with an annotated image of the surgical specimen (middle) and an annotated digitised image of the KRAS G12A (red) and KRAS WT (yellow) subclones visualised by BaseScope in situ hybridisation (right). +, bootstrap value of 65–95; ++, bootstrap value <65; HGD, high-grade dysplasia; HI, homoplasy index; LGD, low-grade dysplasia; MSI, microsatellite instability; WT, wild type.

To probe the temporal sequence of cancer evolution, we constructed phylogenetic trees that showed the order of SNA acquisition for each of the nine multiregion sequenced CA-CRCs (figure 2; eight MSS, one MSI; samplewise mutation presence/absence data in online supplementary table 10). The trees showed the mutational field shared between the cancer and surrounding mucosa (median 21 clonal SNAs per case, range 3–33, online supplementary figure 4A). An average of 16% of the mutations present in all regions of the CA-CRC (truncal mutations) were also found in the surrounding mucosa (range 0.3%–36.3%, online supplementary figure 4B).

10.1136/gutjnl-2018-316191.supp5 Supplementary data

Generally, the phylogenies had both long trunks (large number of clonal SNAs) and long branches (large number of subclonal SNAs), indicating the major carcinoma lineages were relatively genetically distinct from one another. In all but one case, the phylogenetic branches of the carcinomas were also roughly of equal length, indicating that there was no disparity of mutational burden (a potential indicator of subclonal selection, as a faster dividing clone would accumulate mutations at a faster rate) in subregions of the carcinoma. We noted that the conventional driver mutations (*TP53*, *KRAS*, *APC*) were clonal within carcinomas (with the exception of UC06). Together, these data indicate evolutionary dynamics consistent with the ‘Big Bang’ model postulated for S-CRCs,^31^ where a cancer is formed with all the major driver mutations, rather than acquiring them sequentially after the initiation of cancer growth.

UC06 showed an interesting polyclonal architecture (figure 2). In this case, every carcinoma sample, and a surrounding region of HGD contained a clonal *TP53* mutation, but the carcinoma was composed of spatially interwoven but genetically distinct clones bearing *KRAS* and *APC* mutations, respectively. The *APC* mutant clone had also developed an *ARID1A* mutant subclone (figure 2; *ARID1A* detected in a single region of carcinoma). To confirm the presence of a *KRAS* mutant subclone we performed *in situ* mutation detection using BaseScope. We found the *KRAS* mutant subclone was histologically indistinguishable from the *KRAS* wild-type (*APC* mutant) subclone, with the subclones demonstrating spatial restriction, yet with considerable intermixing at the clone boundary (figure 2). These data confirm that *APC* mutation did not play a gatekeeping role in the development of this carcinoma.

We examined the phylogenetic location of SNAs within our 16 putative CA-CRC driver genes and found that they are often truncal (‘field mutations’, 9/30 mutations, 30%) or clonal within the cancer (9/30, 30%) indicating that mutations in putative CA-CRC genes are generally early events in tumorigenesis. However, they can also be found mutated only in non-dysplastic mucosa (9/30, 30%) or more rarely within a subregion of the CA-CRC (3/30, 10%).

### Mutational signatures in CA-CRCs

Different mutational processes can give rise to distinct patterns of mutations across the genome termed ‘signatures’^32^ (where the patterns are defined by the relative frequencies of substitution type within the sequence context immediately 3′ and 5′ to the mutated base). To investigate the mechanistic processes that drive mutation acquisition in the inflamed colon we used WXS data to construct mutational signatures (n=12 CA-CRCs; see online supplementary figure 5 for individual carcinoma signatures and online supplementary figure 6A for the composite 96-channel signature of all carcinomas). MSS signatures were mostly composed of signature 1 (median 66.9%, online supplementary figure 6B), which represents spontaneous deamination of 5-methylcytosine (an ageing-associated signature). There was no significant difference in the median contribution of signature 1 in CD cases (70.7%) and UC cases (63.9%, p=0.83). In MSS CA-CRC we also detected a considerable contribution of signature 5 (median 10.9%), a common cancer-associated signature of unknown aetiology that is also associated with ageing. As expected, the signatures of MSI cases UC02 and UC09 are largely dominated by signature 6 (defective DNA mismatch repair; online supplementary figure 6B).

The mutational signature regressions using the chosen set of signatures gave an average fit (mean R^2^) of 78% across CA-CRC samples (range 50%–90%). The fits were not significantly different from those for S-CRC (p=0.14; mean R^2^ 83%, range 14%–99%). These results suggest that there are unlikely to be additional mutational processes that are highly active in the CA-CRC data that are not also active in the S-CRC data. However, the possibility that other mutational processes are active cannot be ruled out.

We performed analysis of precancer, truncal (clonal) and branch (subclonal) SNAs separately, and found no significant difference in the relative or absolute contribution of any signature (online supplementary figures 6C and D), providing no evidence of large alterations in mutational process activity between the precancerous colitic bowel, and early and late in cancer progression. Comparison of the mutational signatures of CA-CRCs to their S-CRC counterparts (TCGA data set^23^) showed that CA-CRCs tended to have slight (but not statistically significantly different) increases in the absolute and relative contribution of most signatures (online supplementary figures 6E and F).

### Extensive CNAs in CA-CRCs

**Figure 3:**
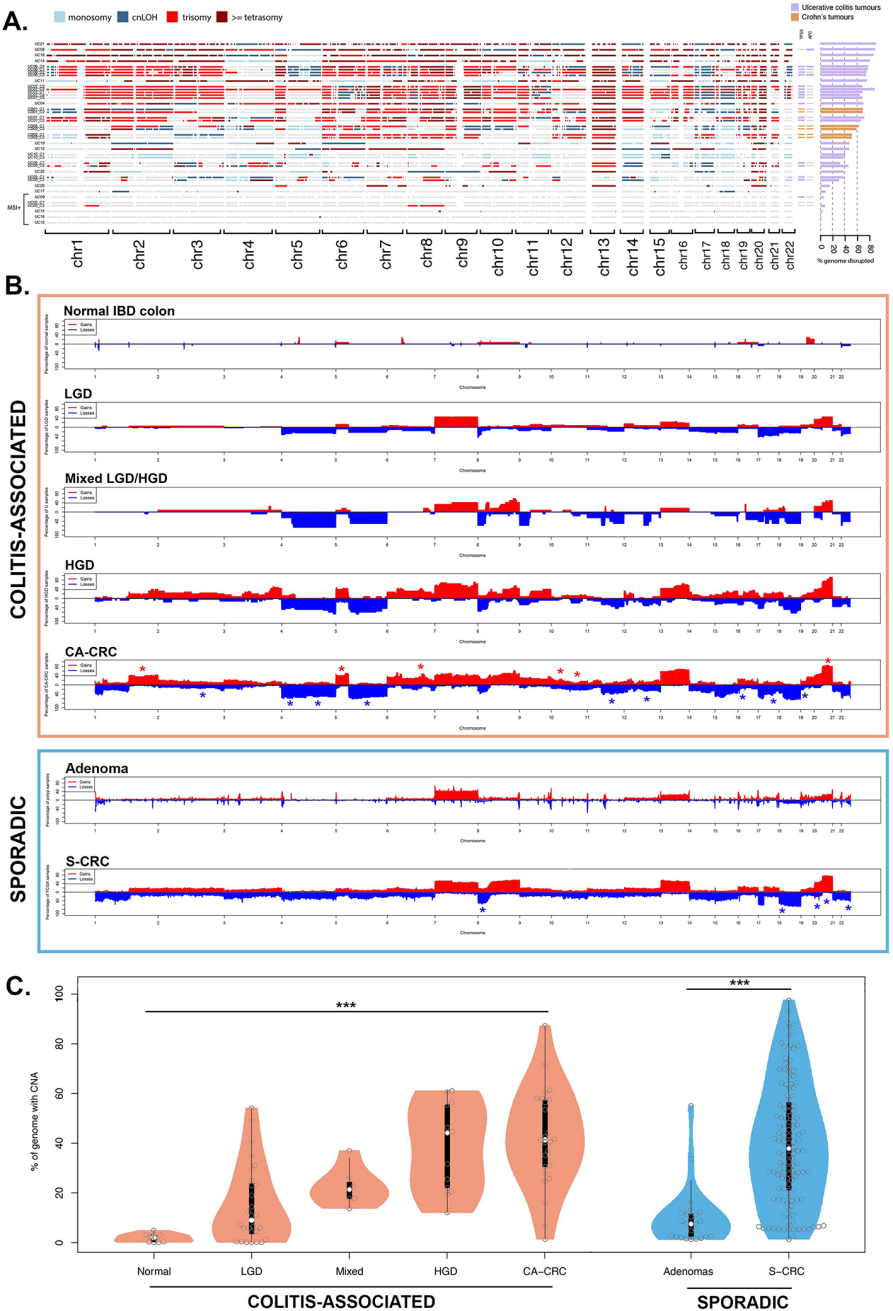
Analysis of genome-wide CNAs in CA-CRC. (A) Per sample analysis of CNAs (copy gain, copy loss or copy-neutral loss of heterozygosity (LOH), derived from whole exome sequencing (WXS) or single nucleotide polymorphism (SNP) array) in microsatellite stable (MSS) and microsatellite instable (MSI) CA-CRCs, represented as a genome-wide plot (left panel) and as a proportion of the genome disrupted (right panel). Also shown is the mutational status of the *TP53* and *APC* genes in each case (coloured box=mutant, grey box=wild type). (B) Genome-wide frequency of losses and gains in normal IBD colon, dysplasia (LGD, mixed LGD/HGD, HGD), MSS CA-CRC, sporadic adenomas and MSS S-CRC. Red or blue crosses indicate statistically significant arm-level amplification or loss, respectively. Normal IBD colon: n=9, LGD: n=28, mixed LGD/HGD: n=7, HGD: n=13, CA-CRC: n=25, sporadic adenoma: n=25, S-CRC: n=127. (C) Analysis of the proportion of the genome showing loss or gain in normal IBD colon, dysplasia, CA-CRC, sporadic adenoma and S-CRC (for colitis-associated samples p=3.14×10^−8^ by the Kruskal-Wallis test; for sporadic samples p=3.43×10^−9^ by the Mann-Whitney test). Sample numbers were as described in (B). CA-CRC, colitis-associated CRC; CNA, copy number alteration; cnLOH, copy-neutral LOH; HGD, high-grade dysplasia; LGD, low-grade dysplasia; S-CRC, sporadic CRC.

We analysed the genome-wide allelic CN profiles of the 13 carcinomas with WXS data and of a further 12 FFPE carcinomas assayed by SNP-array alone (n=25 carcinomas total; figure 3A). To validate selected individual chromosomal CN change, fluorescence *in situ* hybridisation was performed in a subset of carcinomas (n=8; online supplementary figure 7 and supplementary table 11).

MSS CA-CRCs (n=20) had extensive CNAs, with a median of 64.9% (range 8.3%–90.9%) of the genome showing either copy loss, copy gain or copy-neutral loss of heterozygosity (LOH). There was no significant difference between CNA events in Crohn’s and patients with UC, with CD and UC cases showing similar proportions of the genome altered (61.0% vs 68.8%, p>0.99 by the Mann-Whitney test). We noted that large-scale genomic alterations are common in CA-CRC; four MSS carcinomas (20%) appeared tetraploid or near tetraploid, and a further eight MSS carcinomas (40%) appeared triploid or near triploid, features which did not appear to correlate with mutation of *TP53* or *APC* (figure 3A). Five cases were identified as MSI, and these displayed low levels of CNAs (median 3.0% genome altered, range 0.7%–5.4%). Online supplementary table 12 provides the CN status of common CRC driver genes for each sample. It is notable that 5q22.2 (*APC*) allelic loss is more common in CA-CRC (9/20 cases, 45%) than in MSS S-CRCs (42/189, 22%, TCGA data,^23^ p=0.051 by Fisher’s test), perhaps indicating that Wnt signalling in CA-CRCs is disrupted by copy loss of *APC* rather than mutation.

To analyse which CNAs were altered specifically in CA-CRCs, we supplemented our WXS and SNP array data with LP-WGS data (n=7 CA-CRCs). This methodology detects copy-loss and copy-gain events but cannot resolve copy-neutral LOH (cnLOH) events. MSS CA-CRCs had a distinct profile of losses and gains as compared with MSS S-CRC (figure 3B; comparison with TCGA data^23^). At arm level, six chromosomal arms were significantly more likely to be gained in CA-CRC compared with S-CRC and 10 arms were significantly more likely to be lost, most notably 5q (57% vs 17%; OR=5.87; *q*<0.001) and 17q (37% vs 15%; OR=3.34; *q*=0.01, online supplementary table 13). Furthermore, there were five chromosomal arms that were less likely to be lost in CA-CRC (online supplementary table 13). These genomic differences were despite S-CRC and CA-CRC having a similar proportion of the genome with losses or gains (excluding cnLOH events: 37.9% vs 41.2%, p=0.6, figure 3C). LGD colitis-associated lesions had similar proportions of the genome lost or gained to sporadic adenomas (7.5% vs 10.7%, p=0.4; n=25 sporadic adenomas, assayed by SNP array), but colitis-associated lesions of higher grade tended to have a significantly higher proportion of the genome with losses or gains (mixed LGD/HGD: 21.2% vs 10.7%, p<0.003; HGD: 44.0% vs 10.7%, p<0.001, figure 3C).

### Evolutionary history of CNAs

The data above indicated a potentially critical role for CNAs in the progression from benign to malignant disease in colitis. To map precisely when CNAs occurred during progression, we performed a cross-sectional analysis of CNAs in additional non-dysplastic mucosa (n=9), LGD (n=28), mixed LGD/HGD (n=7), HGD (n=13) and compared these data to our MSS CA-CRCs (figure 3B,C and online supplementary table 14). Non-dysplastic mucosa rarely contained CNAs, and if present they affected only a small percentage of the genome (median 1.9%, range 0%–4.7%). LGD had significantly increased levels of CNAs (median 8.9%, range 0%–53.8%, p=0.006, figure 3C), with the most common alterations being gain of Chr7 (45%) and gain of Chr20q (45%; figure 3B). HGD had a much elevated CNA burden relative to LGD (median 44.0%, range 12.0%–60.8%, p=0.0002; figure 3C) and many of the alterations evident in LGD were also evident in HGD, including the Chr7 and Chr20q gains. HGD and CA-CRC (median 41.2%, range 1.3%–86.9%) had similar total CNA burdens. These data show a progressive increase in CNA burden during progression, with a large increase in burden acquired at the LGD to HGD transition, where there is limited further evolution of CN. Together, these data point to a common and critical central role for CNAs in the development of CA-CRC.

**Figure 4:**
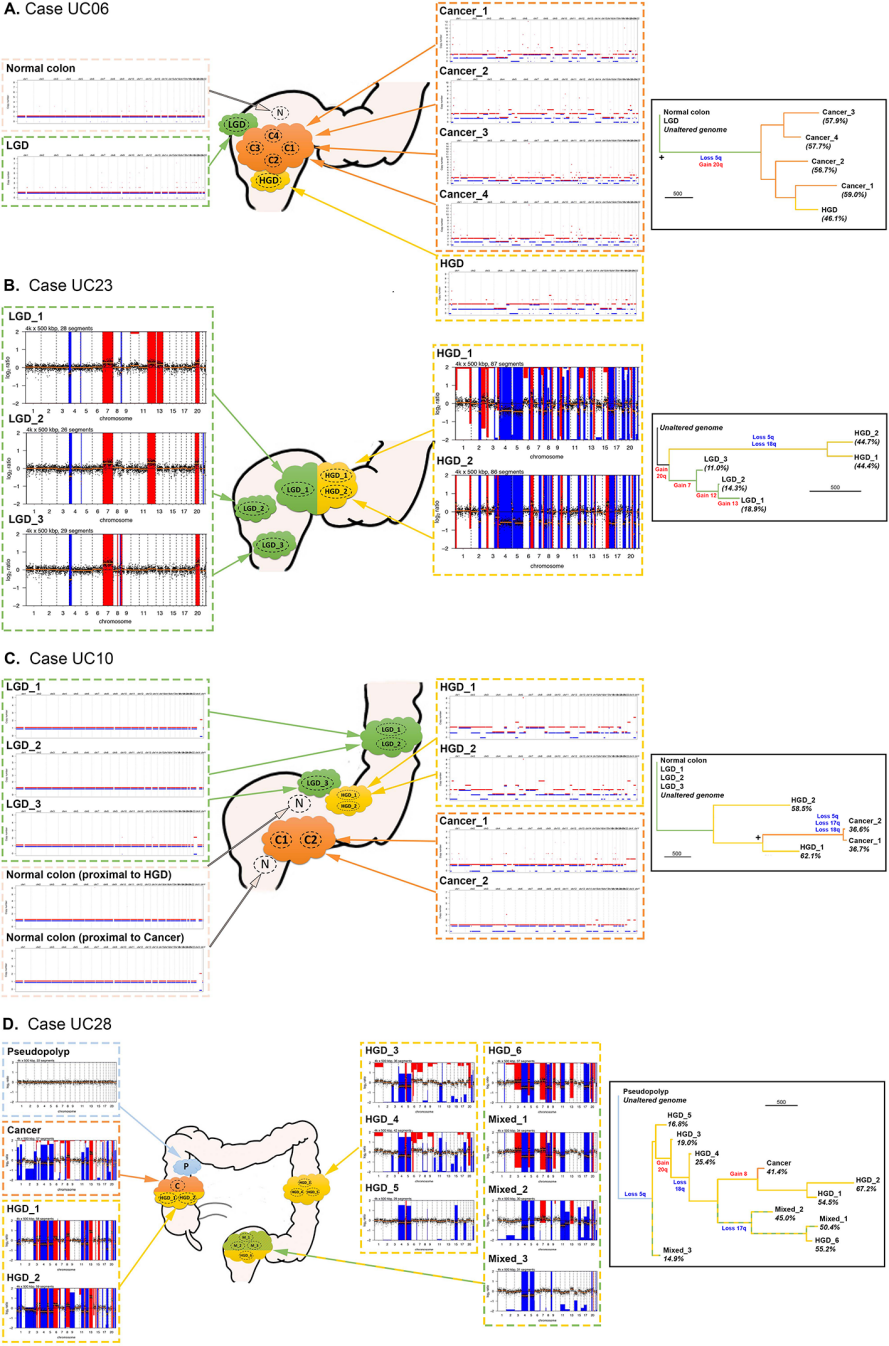
Analysis of copy number alteration (CNA) phylogeny in colitis-associated CRC (CA-CRC). Left panels show representative schematics of the location of biopsies, annotated with genome-wide copy number (CN) plots derived from whole exome sequencing (WXS) (A, C) or low-pass whole genome sequencing (LP-WGS) (B, D). Right panels show phylogenetic trees produced using the corresponding CN data, with key CN gains and losses annotated. + indicates bootstrap value of 65–95. The scale bar for branch length represents 500 evolutionary changes of bin size 500 kbp (see the Methods section). HGD, high-grade dysplasia; LGD, low-grade dysplasia.

To gain insight into the temporal accrual of CNAs, we built phylogenetic trees using CNA data alone (figure 4). Key arm-level CNAs of interest (derived from figure 3B) were manually annotated onto the phylogenetic trees using the CNA events visible on individual genome-wide CN plots. We observed considerable heterogeneity in the phylogenies of the CNA trees. Case UC06 (figure 4A) showed a long ‘trunk’ separating LGD from HGD/CA-CRC, with short branches representing each HGD/CA-CRC biopsy. This indicates a large evolutionary distance between LGD and HGD/CA-CRC, and little continued CNA evolution of HGD/CA-CRC: a pattern indicative of punctuated genetic evolution where CNAs accrue and stabilise in the population in ‘bursts’ rather than continuously over time. A similar relationship was observed between the LGD and HGD biopsies of case UC23 (figure 4B), again indicating a punctuated evolution of CN state, with a large accrual of CNAs occurring at the transition from LGD to HGD. In other cases, there was evidence of ongoing genomic instability in HGD: UC10 showed considerable evolution of CN state after the HGD transition (figure 4C), indicated by the relatively short trunk, and long branches of the HGD biopsies. Case UC28 (figure 4D) also showed a large amount of CN evolution occurring within HGD, with some regions of HGD even containing a higher proportion of genomic alterations than the CA-CRC. Thus, although the proportion of CNAs distinguishes LGD and HGD, the resulting CN state is not always stable, and HGD/CA-CRC can display striking chromosomal instability.

## Discussion

CA-CRCs are molecularly distinct from their sporadic counterparts: CA-CRCs have an increased burden of SNAs compared with S-CRCs, and these include recurrent mutations in genes infrequently mutated in S-CRCs and differential mutation frequency of genes commonly mutated in both tumour types. Moreover, CA-CRCs tend to have a high burden of CNAs with recurrent losses and gains distinct from S-CRCs. Here we have used multiregion genomic analysis to reveal the evolutionary history of these differences. From a clinical perspective, understanding the origins of these genetic differences offers a route to precision molecular diagnosis, prevention and treatment of colitis-associated lesions.

Previous NGS studies of CA-CRC used WXS of FFPE CA-CRCs (n=33),^19^ and targeted sequencing of ~300 genes in FFPE CA-CRCs (n=47).^20^ Our WXS of fresh-frozen material revealed a mutation burden of 3.0/Mb, significantly higher than that reported for FFPE material^19^ (1.33/Mb, p<0.0001), presumably because of the necessity of stringent variant filtering in the previous study.^33^ Nevertheless, we note that both WXS studies report elevated ‘accelerated ageing’ mutational processes, as evidenced by an excess of C>T transitions in CA-CRC. The three NGS studies report different mutation frequencies for key genes, most likely because of the relatively small sample sizes of each cohort. We suggest that our meta-analysis of these three studies (n=87; figure 1D) should provide the most accurate measurement of gene somatic mutation frequency in CA-CRC.

**Figure 5:**
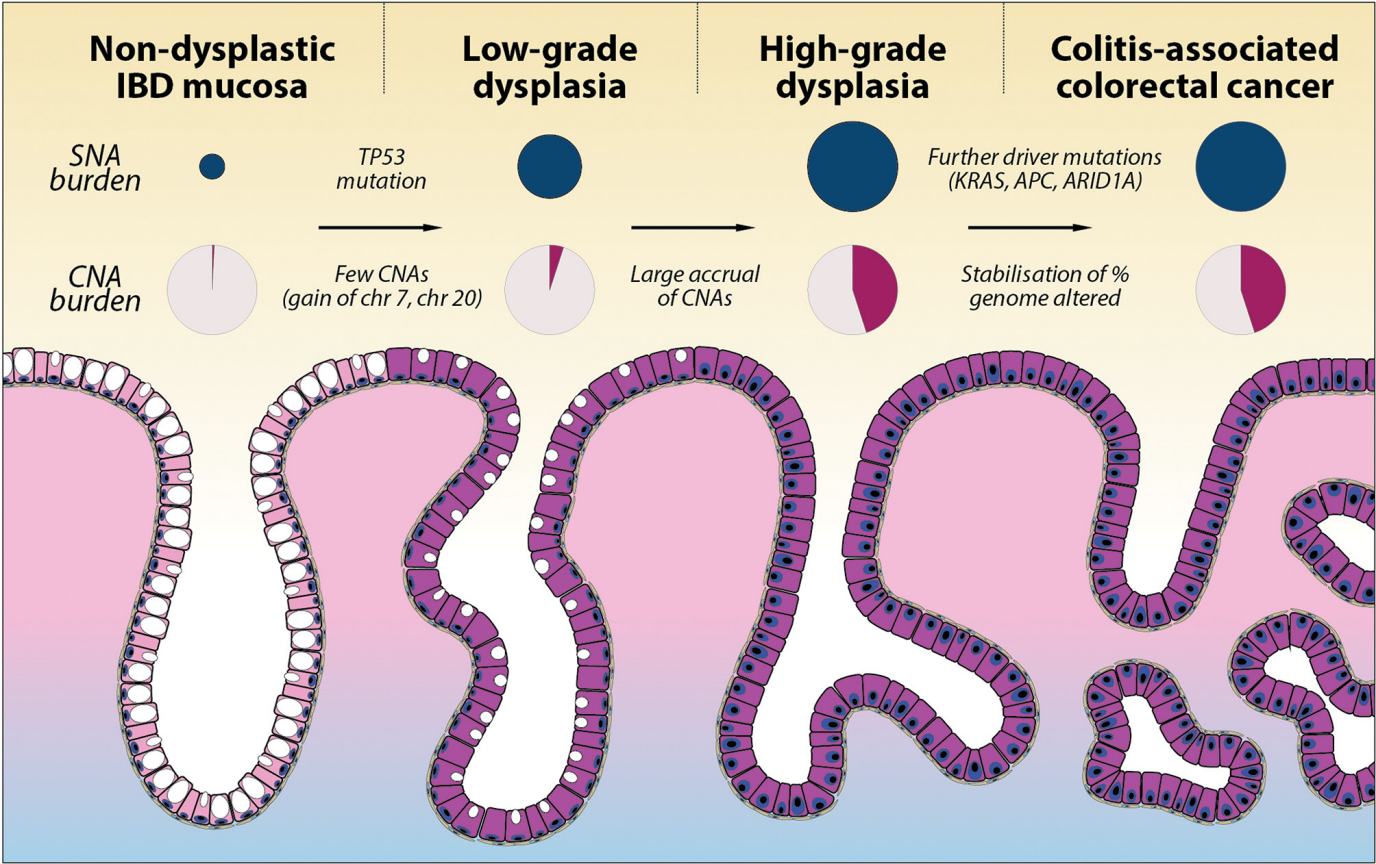
Summary of SNA and CNA burden during colitis-associated CRC (CA-CRC) tumorigenesis. Schematic diagram showing the stepwise progression to CA-CRC, annotated with the observed SNA burden (proportional to the size of dark blue circles) and CNA burden (shaded area of pink circles). CNA, copy number alteration; SNA, single nucleotide alteration.

Our data reveal that the high SNA burden of CA-CRCs began accruing prior to cancer formation, in the non-dysplastic epithelium. Remarkably, the mutation burden of non-dysplastic tissue was as high as that of the established CA-CRC in a third of cases. Mutational processes were dominated by ageing-associated signatures. This implies that the inflammation and injury-induced cell turnover that is required for intestinal repair and restitution comes with a potentially attritional biological ‘cost’ of mutation acquisition. Nevertheless, the mutations generated by this ‘accelerated ageing’ process are inevitably subject to natural selection imposed by the microenvironment of the colitic bowel, leading to the emergence of a unique mutational composition of neoplastic lesions that arise from the field.^21^ *TP53* mutations were always present at clonal frequency in CA-CRCs (consistent with previous reports^10–12^), confirming that it is universally an early event in colitis-associated tumorigenesis (figure 5). Mutations in other conventional drivers (*APC*, *KRAS*, *PIK3CA*, *ARID1A*) were usually clonal within a carcinoma. On examining the clonality of 16 genes that were significantly more frequently mutated in CA-CRC we found that 60% were shared with the surrounding normal mucosa, or mutated at clonal frequency in the carcinoma, indicating they are common early events. Together, these data add to our previous candidate gene demonstration of field cancerisation^11 34^ by showing the extent of mutational burden present in surrounding non-dysplastic mucosa. This highlights the role of field cancerisation in the aetiology of CA-CRC and suggests that particular cellular phenotypes induced by somatic mutation undergo positive selection in the inflamed colon.^35^ These data are suggestive that assaying the mutations present in the inflamed bowel could provide an objective measure of the risk of colitis-associated cancer development.

CNAs also begin to accrue prior to cancer formation, with non-cancerous HGD lesions notably having a similar overall burden and frequency of particular chromosomal losses and gains as CA-CRCs. Aneuploidy is well known to precede or co-occur with dysplasia in CA-CRC,^15 16 36^ and fields of small region (<1 Mb) CN changes have been detected in the majority of patients with UC, particularly in those who progress to CA-CRC.^37^ Presumably, aneuploidy allows for rapid acquisition of adaptive phenotypes to enhance fitness and promote cell survival in the inflamed colon. Further work is needed to identify which loci are under selection on recurrently lost and gained chromosomes. Aneuploidy may also be a ‘side effect’ of p53 mutation, with the latter potentially associated with a survival advantage in colitis. Importantly, our data show a typical ‘sudden’ increase in CNA burden at the transition from LGD to HGD (figure 5). From a clinical perspective, this stark molecular distinction between LGD and HGD suggests a route to objective molecular-based pathological diagnosis of tissue grade, though the apparent sudden ‘punctuated’ accrual of CNAs may imply a limited window for early detection of high-risk LGD lesions.

Our data are suggestive that CA-CRCs evolve according to the ‘Big Bang’ model proposed for S-CRC evolution.^31^ We observed that (known and candidate) driver mutations were almost exclusively present in the founder lineage of the tumour, the majority of CNAs occur prior to the onset of cancer growth, and phylogenetic tree shape analysis provided no evidence of differential selection between lineages, though the power of this latter analysis is limited. Together, these data are consistent with the notion that the final tumour expansion is initiated by a particularly evolutionarily fit clone, the expansion of which dominates the final tumour and attenuates the expansion of other (potentially marginally fitter) clones. Consequently, within tumour dynamics appear effectively neutral.^38 39^

In summary, our data show that the evolutionary trajectory of CA-CRC begins long prior to the development of CRC, in a hotbed of mutated, field cancerised, inflamed mucosa. While there are clear genetic differences between S-CRCs and CA-CRCs, there are also broad similarities with both cancer types having recurrent mutations in key CRC driver genes including *TP53*, *KRAS* and *ARID1A*, and showing evidence of similar mutational processes. Chronic inflammation does not appear to be mutagenic *per se*, but instead it accelerates mutation accrual and provides a distinctive selective pressure. Finally, the early specification of the mutational make-up of colitis-associated lesions supports ongoing research into the development of precision molecular panels for the diagnosis of true colitis-associated dysplastic lesions, and their stratification by cancer risk.
