# Supplementary material for: Evolutionary history of human colitis-associated colorectal cancer
Source: Gut. 2018 Jul 10;68(6):985–95. doi: 10.1136/gutjnl-2018-316191 (PMC6580738; doi:10.1136/gutjnl-2018-316191)
Supplement: Supplementary data [file gutjnl-2018-316191supp001.pdf]

Supplementary Table 1 – Clinical characteristics of CA-CRC patients used for WES

	IBD	Age	Sex	Tumour site	Samples sequenced	Colitis duration (years)	Disease extent	PSC	Histology	Stage	MSI
**UC01**	UC	30	M	Hepatic flexure	- Tumour (x2)<br>- Pseudopolyp (x2)<br>- Normal mucosa<br>- Muscle	9	PC	No	Moderate, mucinous	T3N0 M0	No
**UC02**	UC	72	M	Splenic flexure	- Tumour (x2)<br>- Normal mucosa (x2)<br>- Blood	23	PC	Yes	Poor	T3N0 M0	Yes
**UC03**	UC	43	M	Sigmoid	- Tumour (x2)<br>- Normal mucosa<br>- Blood	28	PC	No	Moderate	T1N1 M0	No
**UC04**	UC	53	F	Rectum	- Tumour<br>- Blood	≥10	U	No	Moderate	T1N0 MX	No
**UC05**	UC	60	M	Sigmoid	- Tumour (x2)<br>- Normal mucosa<br>- Blood	14	PC	No	Poor	T3N2 M1	No
**UC06**	UC	71	M	Rectum	- Tumour (x4)<br>- HGD<br>- LGD<br>- Normal mucosa<br>- Blood	21	PC	No	Well	T2N0 M0	No
**UC07**	UC	36	M	Caecum	- Tumour (x5)<br>- LGD<br>- Normal mucosa (x3)<br>- Blood	14	PC	Yes	Poor, mucinous	T3N2 M0	No
**UC08**	UC	45	M	Sigmoid	- Tumour<br>- Blood	≥10	U	No	Moderate	T3N0 M0	No
**UC09**	UC	78	U	Rectum	- Tumour<br>- Blood	≥10	PC	No	Well	T3N1 M1	Yes
**UC10**	UC	36	M	Sigmoid	- Tumour (x2)<br>- HGD (x2)<br>- LGD (x3)<br>- Normal mucosa (x2)<br>- Blood	11	PC	No	Moderate, mucinous, signet ring	T2N0 M2	No
**CD01**	CD	31	F	Rectum	- Tumour (x2)<br>- Normal mucosa<br>- Blood	≥10	U	Yes	Moderate	T2N0 M0	No
**CD02**	CD	66	F	Caecum	- Tumour (x2)<br>- Normal mucosa<br>- Blood	10	PC	No	Moderate	T2N0 M0	No
**CD03**	CD	51	F	Sigmoid	- Tumour (x2)<br>- Normal mucosa<br>- Lymphocytes	≥10	Left-sided	No	Moderate	T2N0 M0	No

UC = ulcerative colitis, CD = Crohn's disease, PSC = primary sclerosing cholangitis, MSI = microsatellite instability, U = unknown, PC = pancolitis

Supplementary Table 2 – Clinical characteristics of CA-CRC patients used for SNP array analysis

	IBD	Disease Extent	Age	Sex	Tumour Site	Stage	Samples arrayed	Colitis duration (years)	PSC	MSI
**UC11**	UC	PC	64	M	Sigmoid	T2N0M0	- Tumour	20	No	No
**UC12**	UC	PC	87	M	Caecum	T1N0M0	- Tumour<br>- Normal mucosa	8	Yes	No
**UC13**	UC	PC	35	M	Rectum	T2N0M0	- Tumour<br>- Normal mucosa	11	No	Yes
**UC14**	UC	PC	29	F	Rectosigmoid	T2N1M0	- Tumour	12	No	No
**UC15**	UC	PC	74	F	Caecum	T2N0M0	- Tumour<br>- Normal mucosa	17	No	Yes
**UC16**	UC	PC	86	F	Ascending	T1N0M0	- Tumour	23	No	Yes
**UC17**	UC	PC	72	M	Caecum	T2N1M0	- Tumour<br>- Normal mucosa	17	No	No
**UC18**	UC	PC	49	F	Caecum	T1N0M0	- Tumour	≥ 10	No	No
**UC19**	UC	PC	60	F	Sigmoid	T3N0M0	- Tumour<br>- Normal mucosa	29	No	No
**UC20**	UC	PC	47	M	Sigmoid	T3N0M0	- Tumour<br>- Normal mucosa	20	No	No
**UC21**	UC	PC	62	M	Sigmoid	T1N0M0	- Tumour	≥ 10	No	No
**UC22**	UC	PC	67	M	Descending	T1N0M0	- Tumour<br>- Normal mucosa	≥ 10	No	No

UC = ulcerative colitis, PSC = primary sclerosing cholangitis, MSI = microsatellite instability, U = unknown, PC = pan-colitis

Supplementary Table 3 – Clinical characteristics of CA-CRC patients used for LP-WGS

	IBD	Disease Extent	Age	Sex	Neoplastic lesion location	Samples sequenced	IBD duration (years)	PSC	MSI
**UC23**	UC	PC	73	F	Rectum (x3)	- HGD (x2)<br>- LGD (x3)	53	No	No
**UC24**	UC	PC	42	M	Ascending	- Cancer (T2N0M0)	12	No	No
**UC25**	UC	PC	71	F	Rectum	- HGD (x3)<br>- LGD	45	Yes	No
**UC26**	UC	PC	62	M	Rectum (x3)	- LGD (x6)	14	No	No
**UC27**	UC	PC	62	M	Rectum (x3)<br>Sigmoid<br>Descending	- HGD (x3)<br>- Mixed LGD/HGD<br>- LGD (x6)	16	No	No
**UC28**	UC	PC	65	M	Ascending (x2)<br>Descending<br>Rectum	- Cancer (T2N0M0)<br>- HGD (x6)<br>- Mixed LGD/HGD (x3)<br>- Pseudopolyp	23	No	No
**UC29**	UC	PC	49	F	Ascending (x2)	- Cancer (T1N0M0)<br>- LGD (x3)	38	Yes	Yes
**UC30**	UC	PC	54	M	Rectum	- Cancer (T1N0M0)<br>- LGD (x2)	31	No	No
**UC31**	UC	PC	78	F	Rectum	- Mixed LGD/HGD	12	No	No
**UC32**	UC	PC	47	M	Sigmoid<br>Rectum (x2)	- Mixed LGD/HGD (x4)<br>- LGD	20	No	No
**UC33**	UC	PC	60	F	Rectum	- Mixed LGD/HGD<br>- LGD	29	No	No
**UC34**	UC	PC	64	M	Sigmoid	- HGD (x2)	20	No	No
**UC35**	UC	PC	87	M	Caecum<br>Ascending (x3)	- Cancer (T1N0M0)<br>- HGD<br>- LGD (x3)	8	No	No
**UC36**	UC	PC	63	M	Sigmoid	- HGD (x4)	17	No	No
**UC37**	UC	PC	26	M	Rectum	- LGD (x3)	9	Yes	No
**UC38**	UC	PC	50	M	Rectum	- Cancer (x2)	25	No	No
**UC39**	UC	PC	69	F	Ascending (x3)	- Mixed LGD/HGD (x2)<br>- LGD (x4)	42	No	No
**UC40**	UC	PC	69	F	Ascending<br>Transverse	- HGD<br>- LGD (x4)	25	Yes	No
**UC41**	UC	PC	59	M	Sigmoid	- LGD	28	Yes	No

UC = ulcerative colitis, PSC = primary sclerosing cholangitis, MSI = microsatellite instability, PC = pan-colitis

Supplementary Table 4 – Primer sequences

Primer	Sequence (5’->3’)
KRAS Forward	GAGTTTGTATTAAAAGGTACTGGTGGA
KRAS Reverse	ATCAAAGAATGGTCCTGCAC
p53-exon6 Forward	AGAGACGACAGGGCTGGTT
p53-exon6 Reverse	TGGAGGGCCACTGACAAC
p53-exon7 Forward	TGCTTGCCACAGGTCTCC
p53-exon7 Reverse	GGTCAGAGGCAAGCAGAGG
p53-exon8 Forward	TTTTTAAATGGGACAGGTAGGA
p53-exon8 Reverse	CACCCTTGGTCTCCTCCAC
Bat-25 Forward	TCGCCTCCAAGAATGTAAGT
Bat-25 Reverse	TCTGCATTTTAACTATGGCTC
Bat-26 Forward	TGACTACTTTTGACTTCAGCC
Bat-26 Reverse	AACCATTCAACATTTTTAACCC

Supplementary Table 5 – Summary of SNVs and INDELs from WES

	Biopsy	Non synonymous SNVs	SNV frequency (per megabase)	Total INDELs	INDEL frequency (per megabase)
**UC01**	- Tumour 1	87	2.89	3	0.10
	- Tumour 2	68	2.56	2	0.08
	- Pseudopolyp 1	55	1.79	0	0
	- Pseudopolyp 2	66	2.16	2	0.07
	- Normal mucosa	42	1.37	2	0.07
**UC02**	- Tumour 1	1017	34.56	81	2.75
	- Tumour 2	1026	39.68	82	3.17
	- Normal mucosa 1	24	0.85	12	0.42
	- Normal mucosa 2	74	2.30	9	0.28
**UC03**	- Tumour 1	183	6.02	10	0.33
	- Tumour 2	161	5.53	11	0.38
	- Normal mucosa	42	1.63	1	0.04
**UC04**	- Tumour	70	2.07	5	0.15
**UC05**	- Tumour 1	118	1.15	14	0.14
	- Tumour 2	120	1.19	12	0.12
	- Normal mucosa	114	1.57	10	0.14
**UC06**	- Tumour 1	158	5.20	11	0.36
	- Tumour 2	162	5.36	10	0.33
	- Tumour 3	173	5.61	12	0.39
	- Tumour 4	180	5.92	11	0.36
	- HGD	101	3.31	8	0.26
	- LGD	124	3.98	13	0.42
	- Normal mucosa	79	2.59	11	0.36
**UC07**	- Tumour 1	77	2.44	17	0.54
	- Tumour 2	40	1.63	8	0.33
	- Tumour 3	45	1.60	14	0.50
	- Tumour 4	63	2.13	13	0.44
	- Tumour 5	57	1.99	14	0.49
	- LGD	71	2.23	17	0.53
	- Normal mucosa 1	48	1.61	12	0.40
	- Normal mucosa 2	46	1.43	15	0.47
	- Normal mucosa 3	38	1.25	15	0.49
**UC08**	- Tumour	80	2.38	5	0.15
**UC09**	- Tumour	962	28.72	115	3.43
**CD01**	- Tumour 1	132	4.30	8	0.16
	- Tumour 2	119	3.78	5	0.26
	- Normal mucosa	117	3.82	4	0.13
**CD02**	- Tumour 1	149	4.89	13	0.43
	- Tumour 2	154	4.73	15	0.46
	- Normal mucosa	25	0.80	7	0.23
**CD03**	- Tumour 1	80	3.06	9	0.34
	- Tumour 2	97	3.39	10	0.35
	- Normal mucosa	101	3.64	6	0.22

Supplementary Table 7 – Summary of *TP53* variants in CA-CRC

	**Biopsy**	**TP53 variant**	**VAF(s)**
**UC01**	- Tumour 1<br>- Tumour 2<br>- Pseudopolyp 1<br>- Pseudopolyp 2	- exon8:c.C844T:p.R282W,;exon7:c.G733A:p.G245S; exon7:c.T700C:p.Y234H<br>- exon8:c.C844T:p.R282W,;exon7:c.G733A:p.G245S; exon7:c.T700C:p.Y234H	0.49; 0.42; 0.33<br>0.32; 0.88; 0.88
**UC02**	- Tumour 1<br>- Tumour 2<br>- Normal mucosa 1<br>- Normal mucosa 2		
**UC03**	- Tumour 1<br>- Tumour 2<br>- Normal mucosa	- exon7:c.G733C:p.G245R<br>- exon7:c.G733C:p.G245R	0.24<br>0.47
**UC04**	- Tumour	- exon7:c.G743A:p.R248Q	0.54
**UC05**	- Tumour 1<br>- Tumour 2<br>- Normal mucosa	- exon7:c.675_676G<br>- exon7:c.675_676G	0.36<br>0.52
**UC06**	- Tumour (x4)<br>- HGD<br>- LGD<br>- Normal mucosa	- exon4:c.G202T:p.E68X	0.71, 0.61, 0.67, 0.4<br>0.45
**UC07**	- Tumour (x5)<br>- LGD<br>- Normal mucosa 1<br>- Normal mucosa 2<br>- Normal mucosa 3	- exon6:c.559+1G>A (splice site)<br>- exon8:c.C916T:p.R306X	0.3, 0.4, 0.5, 0.33, 0.45<br>0.38
**UC08**	- Tumour	- exon6:c.376-1G>C (splice site)	0.5
**UC09**	- Tumour	- exon11:c.1146_1147C; exon7:c.560-2A>G (splice site)	0.2, 0.17
**CD01**	- Tumour 1<br>- Tumour 2<br>- Normal mucosa	- exon6:c.C569T:p.P190L<br>- exon6:c.C569T:p.P190L	0.86<br>0.92
**CD02**	- Tumour 1<br>- Tumour 2<br>- Normal mucosa	- exon6:c.C569T:p.P190L<br>- exon6:c.C569T:p.P190L	0.27<br>0.1
**CD03**	- Tumour 1<br>- Tumour 2<br>- Normal mucosa	- exon5:c.G524A:p.R175H<br>- exon5:c.G524A:p.R175H	0.92<br>0.63

Supplementary table 8 – Functional analysis of putative CA-CRC driver genes

Gene	Gene function	Associated pathways	Cancer association	Description
ARID1A	Chromatin remodelling/ transcription	Transcription machinery	Yes	A member of the SWI/SNF family of helicase/ATPases. Regulates gene transcription by altering the chromatin structure.
ASTN2	Neuronal migration	Calcium ion binding	No	Mediates recycling of the neuronal cell adhesion molecule ASTN1 to the anterior pole of the cell membrane in migrating neurons.
CACNA1E	Voltage dependent calcium channel	MAPK signalling, Calcium signalling	Uncertain	Mediates the entry of calcium ions into excitable cells, therefore is involved in a variety of calcium-dependent processes (e.g. gene expression, cell motility, cell division and cell death).
CDC42EP1	Actin cytoskeleton organisation	GTP/Rho binding, actin nucleation, ERK signalling	Uncertain	A CDC42 binding protein that mediates actin cytoskeleton reorganisation at the plasma membrane.
CDH2	Cell-cell adhesion	Cell adhesion molecules	Yes	Encodes N-cadherin (also known as cadherin-2 [CDH2] or neural cadherin [NCAD]), a transmembrane protein that functions to mediate cell-cell adhesion.
CNN2	Cell motility	Actin binding, calmodulin binding	No	Involved in regulating cytoskeleton functions and cell motility.
COL6A3	Structural, collagen	PI3K-Akt signalling Focal adhesion, ECM-receptor interaction	Uncertain	Encodes the alpha-3 chain of type VI collagen, a beaded filament collagen found in most connective tissues.
GUCY1A2	Purine metabolism	Purine metabolism, cGMP-PKG signaling	Uncertain	Catalyses the conversion of GTP to 3',5'-cyclic GMP and pyrophosphate.
GXYLT1	Xylosyltransferase	O-glycan biosynthesis	Uncertain	A glycosyltransferase which elongates the O-linked glucose attached to EGF-like repeats in the extracellular domain of Notch proteins by catalysing the addition of xylose.
PAXIP1	DNA damage response	DNA double strand break repair	Yes	Plays a critical role in maintaining genome stability, condensation of chromatin and progression through mitosis.
POLG	Mitochondrial polymerase	Metabolic pathways, DNA polymerase gamma complex	Yes	The catalytic subunit of the mitochondrial DNA polymerase (DNA polymerase gamma).
PTTG1IP	PTTG1-binding membrane protein	RNA transport, receptor activity	No	A membrane protein, which binds to pituitary tumour-transforming 1 protein (PTTG1), and facilitates its translocation into the nucleus, thereby promoting the transcriptional activation of basic fibroblast growth factor.
RP1L1	Differentiation of photoreceptors	Cytoskeleton, microtubule-binding proteins	No	Binds microtubules and regulates microtubule polymerisation in the retina.
SLC25A5	Mitochondrial ATP/ADP transport	Calcium signalling, cellular senescence	No	Encodes adenine nucleotide translocator 2 (ANT2), a major constituent of the mitochondrial permeability-transition pore complex that catalyses the exchange of mitochondrial ATP with cytosolic ADP.
SLC39A11	Cellular zinc transporter	Solute carrier family, metal ion transporter	No	Increases cytosolic zinc concentrations via uptake across the plasma membrane or efflux from intracellular compartments
TAS2R19	Taste receptor/GI sensing	GPCR activity	No	Involved in the perception of salt and bitter tastes. May play a role in sensing the chemical composition of the gastrointestinal content.

Supplementary table 9 – Meta-analysis of putative CA-CRC driver genes

Gene	Mutant cases CA-CRC (n=10) Current study	Mutant cases CA-CRC (n=30) Robles *et al,* 2016	Mutant cases CA-CRC (n=47) Yaegar *et al,* 2016	Mutant cases S-CRC (n=189)	Fraction mutant in CA-CRC	Fraction mutant in S-CRC	*p* value	Adjusted *p* value (*q* value)
TP53	8	19	42	110	0.79	0.58	0.0007	0.0048
KRAS	4	6	19	82	0.33	0.43	0.1459	0.1772
APC	4	4	10	141	0.21	0.75	2.48E-17	0.0000
SMAD4	0	4	8	18	0.14	0.10	0.3028	0.3418
TAS2R19	5	0	NR	0	0.13	0.00	0.0001	0.0023
FBXW7	3	2	NR	21	0.13	0.11	0.7859	0.8090
ASTN2	3	2	NR	6	0.13	0.03	0.0263	0.0418
TRRAP	0	3	NR	3	0.10	0.02	0.0190	0.0317
SRRM4	0	3	NR	0	0.10	0.00	0.0008	0.0048
RIMS2	0	4	NR	4	0.10	0.02	0.0332	0.0506
NRG1	0	4	NR	3	0.10	0.02	0.0190	0.0317
IL16	0	4	NR	1	0.10	0.01	0.0036	0.0125
DOCK3	0	4	NR	0	0.10	0.00	0.0008	0.0048
COL6A3	4	0	NR	12	0.10	0.06	0.4910	0.5208
CNN2	4	0	NR	0	0.10	0.00	0.0008	0.0048
CACNA1E	3	1	NR	2	0.10	0.01	0.0093	0.0218
ARID1A	3	1	NR	9	0.10	0.05	0.2501	0.2918
PIK3CA	2	3	3	25	0.09	0.13	0.4260	0.4660
SLC39A11	3	0	NR	0	0.08	0.00	0.0050	0.0125
SLC25A5	3	0	NR	0	0.08	0.00	0.0050	0.0125
RRBP1	0	3	NR	0	0.08	0.00	0.0050	0.0125
RP1L1	3	0	NR	4	0.08	0.02	0.1042	0.1351
RADIL	0	3	NR	0	0.08	0.00	0.0050	0.0125
PTTG1IP	3	0	NR	0	0.08	0.00	0.0050	0.0125
POLG	3	0	NR	1	0.08	0.01	0.0175	0.0317
PAXIP1	3	0	NR	0	0.08	0.00	0.0050	0.0125
GXYLT1	3	0	NR	1	0.08	0.01	0.0175	0.0317
GUCY1A2	3	0	NR	3	0.08	0.02	0.0677	0.0911
EP300	0	3	NR	2	0.08	0.01	0.0385	0.0539
CDH2	3	0	NR	5	0.08	0.03	0.1468	0.1772
CDC42EP1	3	0	NR	2	0.08	0.01	0.0385	0.0539
CACNA1D	0	3	NR	1	0.08	0.01	0.0175	0.0317
IDH1	0	0	5	0	0.06	0.00	0.0029	0.0125
BRAF	0	1	1	5	0.02	0.03	1.0000	1.0000
NRAS	1	0	0	17	0.01	0.09	0.0157	0.0317

NR = not reported

Supplementary table 11 - Copy number validation by FISH

Sample	Chr	1	2	3	4	5	6	7	**CN by WES**
CD01	8	3 (2%)	11 (8%)	41 (31%)	20 (15%)	48 (36%)	8 (6%)	1 (1%)	**3**
CD01	18	15 (15%)	22 (21%)	36 (35%)	28 (27%)	2 (2%)	-	-	**2**
CD02	8	7 (7%)	16 (17%)	38 (40%)	35 (36%)	-	-	-	**4**
CD03	5	-	-	1 (1%)	40 (38%)	55 (53%)	8 (8%)	-	**4 / 5**
CD03	8	-	17 (14%)	46 (39%)	44 (37%)	11 (9%)	-	-	**4**
UC02	8	-	18 (18%)	83 (81%)	1 (1%)	-	-	-	**3**
UC05	8	-	25 (71%)	10 (29%)	-	-	-	-	**2**
UC06	5	-	9 (8%)	29 (27%)	17 (16%)	47 (44%)	4 (4%)	-	**4 / 5**
UC06	8	-	1 (1%)	13 (13%)	72 (72%)	5 (5%)	6 (6%)	3 (3%)	**3 / 4**
UC06	18	4 (4%)	30 (34%)	51 (58%)	3 (3%)	-	-	-	**2 / 3**
UC07	5	-	16 (19%)	66 (79%)	1 (1%)	1 (1%)	-	-	**3**
UC07	8	16 (16%)	84 (84%)	-	-	-	-	-	**2 / 3**
UC07	18	29 (30%)	57 (59%)	10 (10%)	1 (1%)	-	-	-	**2 / 3**
UC10	8	-	53 (85%)	9 (15%)	-	-	-	-	**2**
UC10	18	58 (59%)	39 (40%)	1 (1%)	-	-	-	-	**1**

Supplementary table 12 – Copy number analysis of colorectal cancer driver genes

**Gene**	***ARID1A***		***CTNNB1***		***PIK3CA***		***FBXW7***		***APC***		***KRAS***		***TP53***		***ERBB2***	
Locus	1p36.11		3p22.1		3q26.32		4q.31.3		5q22.2		12p12.1		17p13.1		17q12	
Allele	A	B	A	B	A	B	A	B	A	B	A	B	A	B	A	B
CD01_C1	2	0	1	1	1	1	1	1	1	1	20	0	2	0	2	0
CD01_C2	2	0	2	1	1	1	1	1	1	1	8	0	2	0	2	0
CD02_C1	1	0	2	0	2	0	1	1	1	1	2	1	2	1	2	1
CD02_C2	1	0	1	0	1	0	1	1	1	1	2	1	2	1	2	1
CD03_C1	1	1	1	1	2	0	1	0	1	0	2	0	2	0	2	0
CD03_C2	1	1	1	1	2	0	1	0	1	0	2	0	2	0	2	0
UC01_C1	2	2	1	1	4	1	2	0	1	1	2	2	1	1	1	1
UC01_C1	2	1	1	0	3	1	2	1	1	1	2	1	1	1	1	1
UC03_C1	2	0	1	0	2	0	2	0	2	1	2	0	1	1	1	1
UC03_C1	1	0	2	0	1	1	3	0	2	1	2	1	1	1	1	1
UC04_C	1	1	2	0	2	1	2	1	2	2	2	1	1	0	2	1
UC05_C1	1	1	2	0	1	1	2	1	1	1	1	1	1	0	2	0
UC05_C2	1	1	3	0	2	1	2	1	1	1	1	1	1	0	2	0
UC06_C1	2	0	2	2	2	1	1	1	2	0	2	2	2	0	2	0
UC06_C2	2	0	2	2	2	1	1	1	1	0	2	1	2	0	2	0
UC06_C3	2	0	2	2	2	1	1	1	1	0	2	1	2	0	2	0
UC06_C4	2	0	2	2	2	1	1	1	1	0	2	1	2	0	2	0
UC07_C1	1	1	2	1	2	1	1	1	1	1	1	1	2	0	2	0
UC07_C2	2	1	2	1	2	1	2	1	2	1	2	1	2	1	2	1
UC07_C3	1	1	2	1	2	1	1	1	1	1	1	1	2	0	2	0
UC07_C4	1	1	2	1	2	1	1	1	1	1	1	1	2	0	2	0
UC07_C5	1	1	2	1	2	1	1	1	1	1	1	1	2	0	2	0
UC08_C	2	1	2	2	2	2	2	1	2	0	3	2	2	0	2	0
UC10_C1	1	0	1	1	1	1	1	0	1	0	1	1	1	0	1	0
UC10_C2	1	0	1	1	1	1	1	0	1	0	1	1	1	0	1	0
UC11_C	1	0	1	1	0	0	0	1	0	1	3	1	3	1	1	1
UC12_C	1	1	1	1	1	1	1	1	2	0	1	1	2	2	1	1
UC14_C	2	1	2	2	2	2	1	0	1	0	1	1	1	1	1	1
UC17_C	1	1	1	1	1	1	1	1	1	1	1	1	1	1	1	1
UC18_C	12	8	2	2	2	2	2	0	1	1	2	0	2	2	2	2
UC19_C	1	1	1	1	1	1	0	1	0	1	1	1	1	1	1	1
UC20_C	1	1	1	1	1	1	1	1	1	1	1	1	1	1	1	1
UC21_C	1	0	3	1	2	2	4	0	2	1	2	0	2	2	2	2
UC22_C	0	1	1	1	1	1	1	1	1	0	1	1	2	2	2	2

Supplementary Table 13 – CNA frequency in CA-CRC vs. S-CRC

	Fraction in CA-CRC	Fraction in S-CRC	OR	Fisher's Test *p* value	Adjusted *p* (*q* value)
Gain on 1p	0.097	0.030	3.437	0.063	0.174
Loss on 1p	0.274	0.190	1.607	0.211	0.445
Gain on 1q	0.384	0.170	2.892	0.005	0.027
Loss on 1q	0.039	0.100	0.469	0.395	0.701
Gain on 2p	0.178	0.080	2.375	0.077	0.201
Loss on 2p	0.109	0.030	3.437	0.063	0.174
Gain on 2q	0.121	0.100	1.268	0.585	0.801
Loss on 2q	0.150	0.030	5.442	0.005	0.027
Gain on 3p	0.182	0.060	3.402	0.017	0.061
Loss on 3p	0.132	0.090	1.451	0.558	0.777
Gain on 3q	0.107	0.100	0.987	1.000	1.000
Loss on 3q	0.051	0.050	0.988	1.000	1.000
Gain on 4p	0.034	0.010	2.164	0.441	0.717
Loss on 4p	0.554	0.250	3.666	0.000	0.005
Gain on 4q	0.066	0.040	1.997	0.394	0.701
Loss on 4q	0.514	0.240	3.459	0.000	0.008
Gain on 5p	0.428	0.140	4.506	0.000	0.003
Loss on 5p	0.077	0.100	0.721	0.779	0.964
Gain on 5q	0.040	0.070	0.700	1.000	1.000
Loss on 5q	0.547	0.170	5.869	0.000	0.000
Gain on 6p	0.371	0.140	3.662	0.001	0.011
Loss on 6p	0.073	0.070	1.076	1.000	1.000
Gain on 6q	0.290	0.140	2.620	0.019	0.063
Loss on 6q	0.151	0.100	1.565	0.408	0.707
Gain on 7p	0.456	0.470	0.920	0.866	1.000
Loss on 7p	0.045	0.010	4.421	0.136	0.303
Gain on 7q	0.400	0.410	0.965	1.000	1.000
Loss on 7q	0.060	0.010	4.421	0.136	0.303
Gain on 8p	0.301	0.280	1.101	0.851	1.000
Loss on 8p	0.271	0.500	0.380	0.010	0.041
Gain on 8q	0.459	0.460	0.964	1.000	1.000
Loss on 8q	0.001	0.120	0.000	0.012	0.047
Gain on 9p	0.296	0.180	1.961	0.086	0.217
Loss on 9p	0.096	0.080	1.248	0.758	0.964
Gain on 9q	0.275	0.150	2.114	0.068	0.182
Loss on 9q	0.063	0.090	0.825	1.000	1.000
Gain on 10p	0.218	0.050	5.400	0.001	0.011
Loss on 10p	0.154	0.090	1.791	0.250	0.476
Gain on 10q	0.129	0.020	7.118	0.005	0.027
Loss on 10q	0.211	0.130	1.694	0.222	0.456
Gain on 11p	0.141	0.090	1.791	0.250	0.476
Loss on 11p	0.144	0.090	1.791	0.250	0.476
Gain on 11q	0.088	0.070	1.473	0.514	0.743
Loss on 11q	0.361	0.110	4.373	0.000	0.005
Gain on 12p	0.175	0.210	0.798	0.680	0.899
Loss on 12p	0.230	0.090	2.939	0.023	0.073
Gain on 12q	0.132	0.180	0.656	0.503	0.743
Loss on 12q	0.240	0.070	4.393	0.001	0.014
Gain on 13q	0.625	0.560	1.307	0.495	0.743
Loss on 13q	0.019	0.040	0.634	1.000	1.000
Gain on 14q	0.090	0.050	2.079	0.261	0.485
Loss on 14q	0.284	0.300	0.887	0.853	1.000
Gain on 15q	0.011	0.020	0.000	1.000	1.000
Loss on 15q	0.438	0.320	1.743	0.109	0.265
Gain on 16p	0.232	0.180	1.330	0.512	0.743
Loss on 16p	0.181	0.050	3.954	0.010	0.041
Gain on 16q	0.165	0.180	0.973	1.000	1.000
Loss on 16q	0.149	0.060	2.834	0.047	0.141
Gain on 17p	0.036	0.050	0.482	0.702	0.912
Loss on 17p	0.564	0.560	1.061	1.000	1.000
Gain on 17q	0.109	0.120	0.811	1.000	1.000
Loss on 17q	0.374	0.150	3.336	0.002	0.014
Gain on 18p	0.110	0.090	1.130	0.770	0.964
Loss on 18p	0.356	0.610	0.344	0.003	0.024
Gain on 18q	0.014	0.010	2.164	0.441	0.717
Loss on 18q	0.611	0.660	0.768	0.477	0.743
Gain on 19p	0.153	0.110	1.441	0.428	0.717
Loss on 19p	0.165	0.040	5.193	0.003	0.024
Gain on 19q	0.321	0.150	2.680	0.013	0.047
Loss on 19q	0.087	0.050	1.519	0.461	0.733
Gain on 20p	0.447	0.580	0.594	0.128	0.302
Loss on 20p	0.110	0.320	0.238	0.004	0.026
Gain on 20q	0.815	0.720	1.831	0.183	0.396
Loss on 20q	0.005	0.150	0.000	0.004	0.026
Gain on 21q	0.104	0.020	5.547	0.022	0.070
Loss on 21q	0.283	0.220	1.330	0.544	0.771
Gain on 22q	0.048	0.030	1.635	0.629	0.846
Loss on 22q	0.477	0.260	2.557	0.008	0.038

Supplementary Table 14 – CNAs linear regression by grade

Grade	Coefficient	Lower 95% CI	Upper 95% CI
**LGD**	0.1319026	-0.002374498	0.2661796
**U**	0.2043117	0.027711592	0.3809118
**HGD**	0.3656963	0.213739674	0.5176529
**CA-CRC**	0.4353876	0.299164891	0.5716103
