# Supplementary material for: Evolutionary history of human colitis-associated colorectal cancer
Source: Gut. 2018 Jul 10;68(6):985–95. doi: 10.1136/gutjnl-2018-316191 (PMC6580738; doi:10.1136/gutjnl-2018-316191)
Supplement: Supplementary data [file gutjnl-2018-316191supp002.pdf]

Supplementary Materials and Methods

DNA extraction

Fresh-frozen samples: For 8 cases we enriched for epithelial content by laser capture microdissection (LCMD). 6 serial sections of 15-micron thickness were taken onto UV-treated PEN-membrane laser capture slides (Carl Zeiss Microscopy, Gottingen, Germany). Sections were lightly stained with methyl green, and then dehydrated through increasing concentrations of ethanol before LCM of carcinoma cells. For the remaining cases, carcinoma tissue of high cellularity was scraped from serial sections on glass slides, using an annotated H&E as a guide. DNA extraction was performed with the QIAamp DNA micro kit as per manufacturer's guidelines (Qiagen Ltd, Manchester, UK). For the germline controls, DNA was extracted from blood where available, using the DNeasy blood and tissue kit (Qiagen Ltd). Where blood was not available, muscle or uninvolved lymph nodes were laser capture micro-dissected as described above.

FFPE samples: Samples were sectioned onto glass slides and lightly stained with methyl green. A serial annotated H&E was used as a guide for scraping of relevant tissue. DNA was extracted using the High Pure FFPET DNA Isolation Kit (Roche, Burgess Hill, UK). DNA quantity was measured using the Qubit fluorometer (Life Technologies, Paisley, UK) and DNA quality was measured using a multiplex PCR assay[1].

Whole exome sequencing (WXS)

Libraries were prepared from high quality DNA extracted from fresh-frozen tissue using the SureSelect Human All Exon (Agilent, Stockport, UK), Nextera Rapid Capture Exome (Illumina, Cambridge, UK), Nextera Rapid Capture Expanded Exome (Illumina) or SeqCap EZ Exome (Roche) as per manufacturer's guidelines. Libraries were sequenced on the Illumina HiSeq 2000/2500 at the Oxford Genomics Centre to a median depth of 52X (range 25-175X) and a median coverage of 68% at 25X (range 42-92%).

FASTQ files were aligned to the human genome 19 (hg19) using the Burrows-Wheeler Aligner[2]. The resulting sequence alignment map (SAM) files were sorted to produce binary alignment map (BAM) files, and then subsequently marked for duplicates using Picard tools (Broad Institute, Cambridge, MA, USA). The base quality scores were recalibrated using the genome analysis toolkit[3].

SNAs in all samples were called using both BCFtools and Platypus[4], and Indels were called using Platypus. The SNAs and Indels that passed our filters were combined to produce the final variant call set (summarised in Supplementary Table 5, full data in Supplementary Tables 6 and 10), which were subsequently annotated using Annovar[5]. Copy number (CN) states were predicted using the software package Sequenza[6]. To infer information on ancestral relationship between samples we built maximum parsimony phylogenetic trees using the software PAUP[7]. Scripts used to perform this analysis, including filtering steps and parameter values, along with scripts used to perform the evolutionary analyses detailed below are publicly available (https://github.com/BCI-EvoCa/Evo_history_CACRC).

SNP arrays

To deduce CN profiles, DNA extracted from 12 archival CA-CRCs and 25 archival sporadic adenomas was repaired using the Infinium FFPE DNA restore kit (Illumina) and then analysed using the Infinium OmniExpress-24 (v1.1, Illumina) array as per manufacturers guidelines. The logR values and B Allele Frequencies (BAF) were obtained from the Illumina GenomeStudio software and then corrected for GC content bias using the genomic wave script from the PennCNV software suite[8]. ASCAT[9] was used for CN profiling, and these profiles were used to supplement the CN data obtained by WXS, in order to determine regions of recurrent gain or loss in CA-CRC.

Low pass whole genome sequencing (LP-WGS)

DNA extracted from 81 regions from 39 archival lesions of mixed histology (representing 19 UC patients) was used to prepare libraries with the NEBNext Ultra II DNA Library Prep Kit for Illumina (New England BioLabs, Ipswich, MA). Between 4-100ng of input DNA was used and between 7-12 cycles of library amplification. Library quality and quantity were verified using the Agilent TapeStation and the Qubit Fluorometer respectively, before equimolar pooling and sequencing on Illumina's NextSeq 500 (High Output Run, 75bp paired end reads), generating a mean depth of 0.11x (range 0.04x to 0.19x). CN profiles were generated as previously described[10] and used to supplement CN data obtained by WXS and SNP array. Sequencing files were processed using the software pipeline in package *QDNASeq*[10] which filtered for blacklisted regions and loess residuals, and corrected for GC content and mappability with bin sizes 500kbp.

Analysis of recurrent copy number gain or loss

Cytoband coordinates were retrieved from the UCSC Genome Browser database (http://genome.ucsc.edu/)[11] and the mean copy number per cytoband was defined for each sample. Relative copy numbers were obtained by subtracting the estimated median ploidy of each sample, as previously estimated by Sequenza for the WXS samples and ASCAT for the SNP array samples. Copy number alterations for LP-WGS samples were called with *CGHcall* within *QDNAseq.*

Phylogenetics of copy number alterations

CNA phylogenetic trees for were produced by computing the genetic distance between samples. The genome was divided into 4401 bins of size 500kbp (corresponding to the bins used for LP-WGS copy number analyses). Each bin was assigned a call of gain (value ≥ 1), loss (value ≤ -1) or no-change (value 0) relative to median copy number of that sample (using the calls from *CGHcall* for LP-WGS data, or inferred integer copy numbers (described above) for SNP-array and exome data). Phylogenetic trees were computed using maximum parsimony (*pratchet* method) rooted at a relatively unaltered genome (all regions = 0), with branch lengths computed using *acctran* function in R package *phanghorn*. 1000 bootstraps were performed to assess tree robustness.

Analysis of mutational signatures

Mutational signatures comparison to sporadic: For CA-CRCs, total mutations were obtained for each carcinoma (coverage>10x, VAF>0.01 and variant reads>2 in at least one carcinoma region). For S-CRCs, mutations were obtained from TCGA Mutect MAF files downloaded from the genomic data commons (GDC) in August 2017. Previously reported mutational signatures were obtained from http://cancer.sanger.ac.uk/cosmic/signatures/ in April 2017. The complement of mutational processes active in the life-history of each carcinoma sample was inferred by classification of mutations into 96 categories following Alexandrov[12], and the use of non-negative least squares regression, implemented in the R package 'nnls'. For this analysis only mutational signatures 1, 2, 5, 6, 10, 13, and 17 were used for the regression. Mutational signatures were re-scaled to exonic trinucleotide frequencies. Regression coefficients (exposures) were found for each signature in each carcinoma. Coefficients were normalized to one for each carcinoma to find relative exposures.

Temporal evolution of mutational signatures: Mutations present in each carcinoma region were identified (coverage>10x, VAF>0.01 and variant reads>2). Mutations in each carcinoma were then categorized into three groups according to timing ('pre-cancer', 'early' and 'late') depending on their distribution within the carcinoma according to the following: mutations in at least one surrounding normal region were deemed 'pre-cancer', remaining mutations were deemed 'early' if they were present in all cancer regions, and 'late' if they were present in a subset of cancer regions. Mutational signatures were assigned to pre-cancer, early and late mutations of each sample using regression as described above. Mutation sets were excluded from this analysis when there were fewer than 50 mutations for the mutational signature assignments.

Fluorescence *in situ* hybridisation (FISH)

Sections of FFPE tissue at 5-micron thickness were stained using the FISH accessory kit (Dako UK Ltd, Ely, UK) according to the manufacturer's guidelines. Sections were incubated overnight at 37°C with SureFISH probes (Agilent) against Chr5 CEP (cat. no. G100535R-8), Chr8 CEP (cat. no. G101034R-8) or Chr18 CEP (cat no G101074R-8). Sections were counterstained with DAPI, before visualisation on the Ariol system (Leica Biosystems, Milton Keynes, UK). Quantification was performed by counting the number of probes present per whole cell (median of 100 cells per slide).

Immunohistochemistry (IHC)

Immunohistochemistry for β-catenin, MSH6 and PMS2 was performed according to standard methods. Briefly, FFPE sections of 4-micron thickness were dewaxed and rehydrated, before blocking endogenous peroxidase activity by incubation with 3% H_2O_2. Antigen retrieval was performed at 95°C at pH 6.0 (β-catenin and MSH6) or pH 8.0 (PMS2) for 20 minutes. Sections were blocked in 2% goat serum and 1% bovine serum albumin for 1 hour at room temperature (RT), before incubation with

rabbit anti-human β-catenin antibody at 1:4000 dilution (cat. no. AB6302, Abcam plc, Cambridge, UK), mouse anti-human MSH6 (cat. no. GTX62383, GeneTex Inc., Irvine, CA) at 1:500 or rabbit anti-human PMS2 (cat. no. GTX62395, GeneTex Inc.) at 1:50 for 1 hour at RT. Signal amplification was performed by incubating with biotinylated anti-rabbit secondary antibody (Dako) at 1:400 dilution for 30 minutes at RT, and Streptavidin-HRP (Dako) at 1:500 dilution for 30 minutes at RT. Detection was performed by incubating with 3,3'-diaminobenzidine (DAB) for 90 seconds, and sections were lightly counterstained with Gill's haematoxylin before dehydration and mounting.

Microsatellite instability analysis

Microsatellite instability (MSI) status of carcinomas was deduced by sequencing the Bat-25 and Bat-26 microsatellite loci as previously described[13]. Briefly, 2ng of DNA was used as template for a multiplex PCR reaction using fluorescently-labelled primers (Supplementary Table 4), and PCR products were separated by capillary electrophoresis on the 3730 DNA analyser (Applied Biosystems). Where WXS was performed, MSI status was tested bioinformatically using MSI-sensor[14].

Targeted sequencing

To validate driver mutations identified by WXS, targeted sequencing of the relevant regions of *TP53* and *KRAS* was performed on laser capture microdissected carcinoma DNA and normal control DNA. PCR conditions and primer sequences (Supplementary Table 4) were as previously described[15]. Sequencing was performed using the BigDye v3.1 terminator cycle sequencing chemistries, as previously described[15].

Supplementary Materials and Methods References

1. van Beers EH, Joosse SA, Ligtenberg MJ, et al. A multiplex PCR predictor for aCGH success of FFPE samples. Br J Cancer 2006;94:333-7.
2. Li H, Durbin R. Fast and accurate short read alignment with Burrows-Wheeler transform. Bioinformatics 2009;25:1754-60.
3. McKenna A, Hanna M, Banks E, et al. The Genome Analysis Toolkit: a MapReduce framework for analyzing next-generation DNA sequencing data. Genome Res 2010;20:1297-303.
4. Rimmer A, Phan H, Mathieson I, et al. Integrating mapping-, assembly- and haplotype-based approaches for calling variants in clinical sequencing applications. Nat Genet 2014;46:912-918.
5. Wang K, Li M, Hakonarson H. ANNOVAR: functional annotation of genetic variants from high-throughput sequencing data. Nucleic Acids Res 2010;38:e164.
6. Favero F, Joshi T, Marquard AM, et al. Sequenza: allele-specific copy number and mutation profiles from tumor sequencing data. Ann Oncol 2015;26:64-70.
7. Wilgenbusch JC, Swofford D. Inferring evolutionary trees with PAUP*. Curr Protoc Bioinformatics 2003;Chapter 6:Unit 6 4.
8. Diskin SJ, Li M, Hou C, et al. Adjustment of genomic waves in signal intensities from whole-genome SNP genotyping platforms. Nucleic Acids Res 2008;36:e126.
9. Van Loo P, Nordgard SH, Lingjaerde OC, et al. Allele-specific copy number analysis of tumors. Proc Natl Acad Sci U S A 2010;107:16910-5.
10. Scheinin I, Sie D, Bengtsson H, et al. DNA copy number analysis of fresh and formalin-fixed specimens by shallow whole-genome sequencing with identification and exclusion of problematic regions in the genome assembly. Genome Res 2014;24:2022-32.
11. Speir ML, Zweig AS, Rosenbloom KR, et al. The UCSC Genome Browser database: 2016 update. Nucleic Acids Res 2016;44:D717-25.
12. Alexandrov LB, Nik-Zainal S, Wedge DC, et al. Signatures of mutational processes in human cancer. Nature 2013;500:415-21.
13. Berg KD, Glaser CL, Thompson RE, et al. Detection of microsatellite instability by fluorescence multiplex polymerase chain reaction. J Mol Diagn 2000;2:20-8.
14. Niu B, Ye K, Zhang Q, et al. MSIsensor: microsatellite instability detection using paired tumor-normal sequence data. Bioinformatics 2014;30:1015-6.
15. Galandiuk S, Rodriguez-Justo M, Jeffery R, et al. Field cancerization in the intestinal epithelium of patients with Crohn's ileocolitis. Gastroenterology 2012;142:855-864 e8.
