# Supplementary material for: Evolutionary history of human colitis-associated colorectal cancer
Source: Gut. 2018 Jul 10;68(6):985–95. doi: 10.1136/gutjnl-2018-316191 (PMC6580738; doi:10.1136/gutjnl-2018-316191)

Supplementary Figure 1 – Validation of SNAs by LCMD and Sanger sequencing
A. and B. Representative images of laser capture microdissected (LCMD) carcinoma (**A**) and normal (**B**) tissue. Left hand panels represent tissue stained with methyl green before LCMD, and right hand panels represent the same region of tissue after completion of LCMD. Scale bars represent 300 micron.
C. Representative Sanger sequencing of *TP53* (left) and *KRAS* (right) mutations. The upper panels show the germline DNA sequence (blood) and the lower panels show the DNA sequence from microdissected carcinoma cells, with the SNA highlighted in yellow.

Supplementary Figure 1

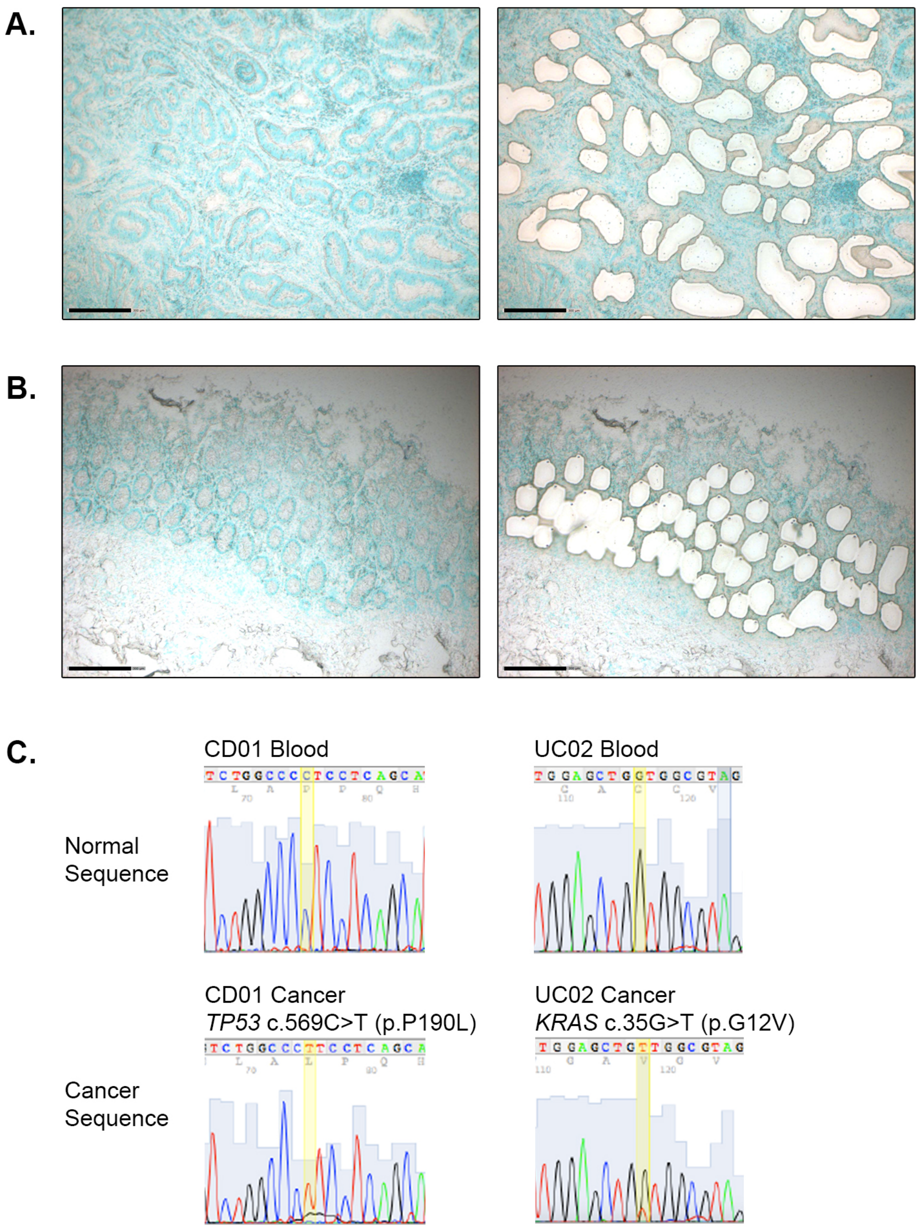

Supplementary Figure 2 – Identification of MSI status

A. Analysis of the Bat-25 and Bat-26 microsatellite loci reveals the MSI status of the UC11 (MSS), UC02 (MSI-positive) and UC15 (MSI-positive) carcinomas. The upper panels (‘Normal’ or ‘Blood’) show the normal alleles with their accompanying ‘stutter’ peaks. In cases UC02 and UC15, the carcinoma samples display novel length alleles, suggesting these loci are positive for MSI. RFU = relative fluorescent units.

B. Representative immunohistochemical staining for MSH6 (upper panels) and PMS2 (lower panels). Scale bars represent 50 micron.

Supplementary Figure 2

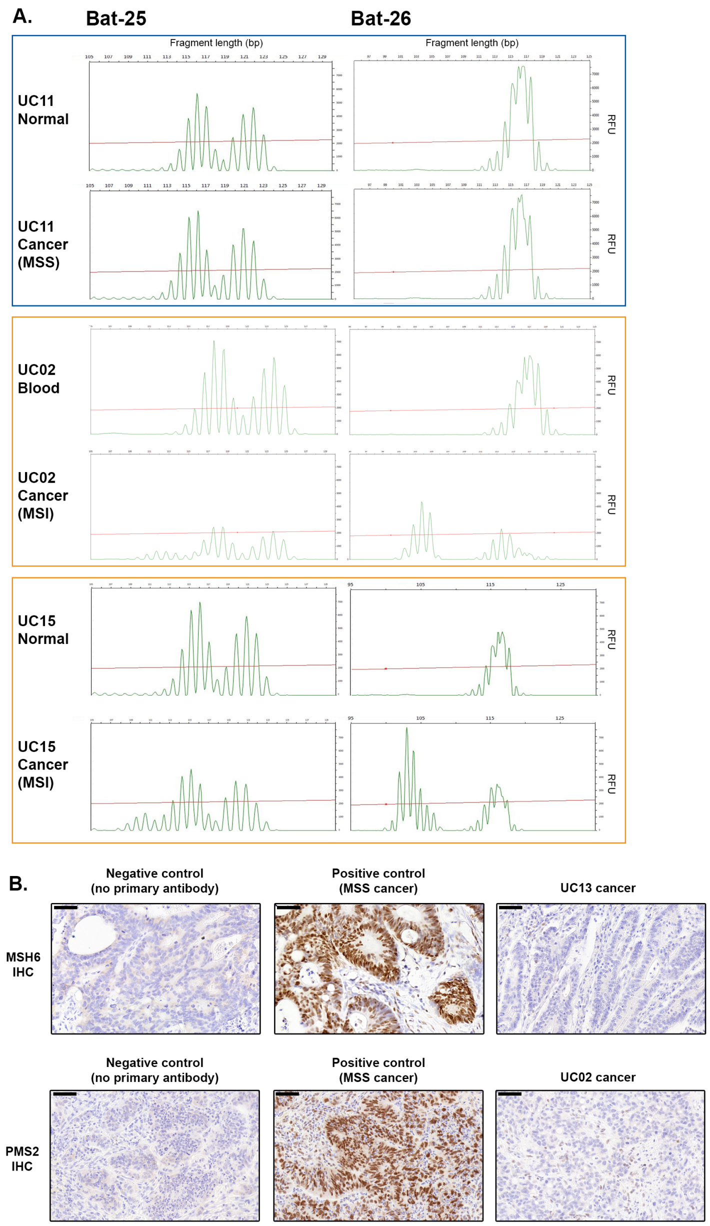

Supplementary Figure 3 – β-catenin expression in CA-CRC and S-CRC

A. Left panel: Quantification of nuclear and cytoplasmic β-catenin expression in CA-CRC (n = 8) and sporadic CRC (n = 13). Right panel: Quantification of nuclear and cytoplasmic β-catenin expression in CA-CRCs with wild type APC (WT, n=5) and CA-CRCs with mutant APC or displaying loss of heterozygosity (LOH) at the APC locus (n=3). *p = 0.024, N.S = not significant by Fisher's test.

B. Representative immunohistochemical staining for β-catenin in CA-CRC. The top panels represent weak nuclear and cytoplasmic staining, and the bottom panels represent strong nuclear and cytoplasmic staining. Scale bars represent 100 micron and 50 micron (inset).

Supplementary Figure 3

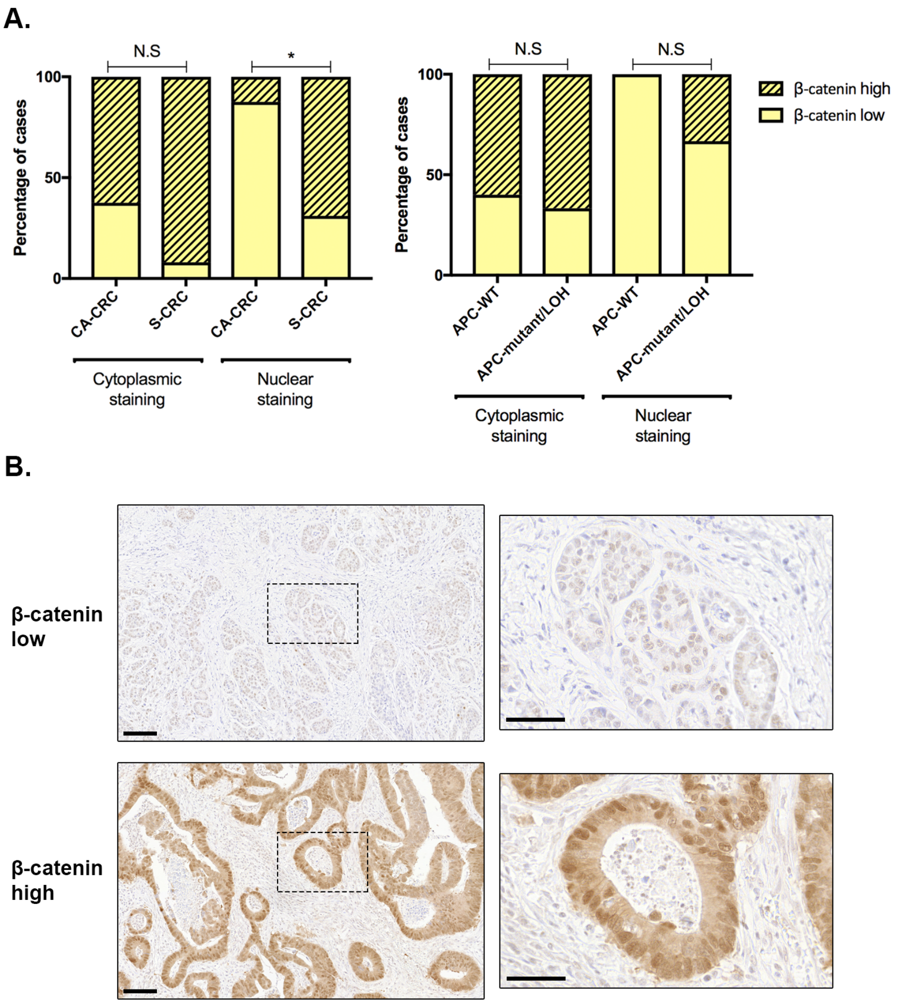

Supplementary Figure 4 – Analysis of the mutational field

A. The number of non-synonymous exonic SNAs that are clonal to the CA-CRC only ('tumour trunk', blue) and those that are also clonal within the surrounding normal mucosa ('mutational field', grey).

B. The number of 'mutational field' mutations as a percentage of the total clonal mutations within each CA-CRC

Supplementary Figure 4

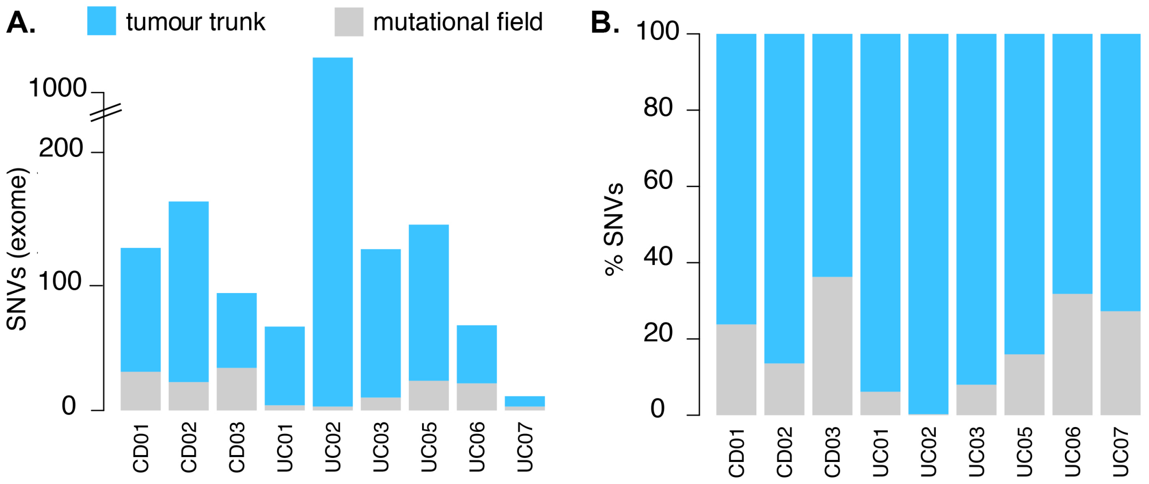

Supplementary Figure 5 – Mutational signature analysis of individual CA-CRCs
Upper plots: 96-channel classification of individual CA-CRCs defined by the substitution class and sequence context immediately 3′ and 5′ to the mutated base. Mutation types are on the horizontal axis and the vertical axis depicts the percentage of mutations attributed to a specific mutation type. Lower plots: Fitted 96-channel classification combining mutational signatures 1, 2, 5, 6, 10, 13, and 17.

Supplementary Figure 5

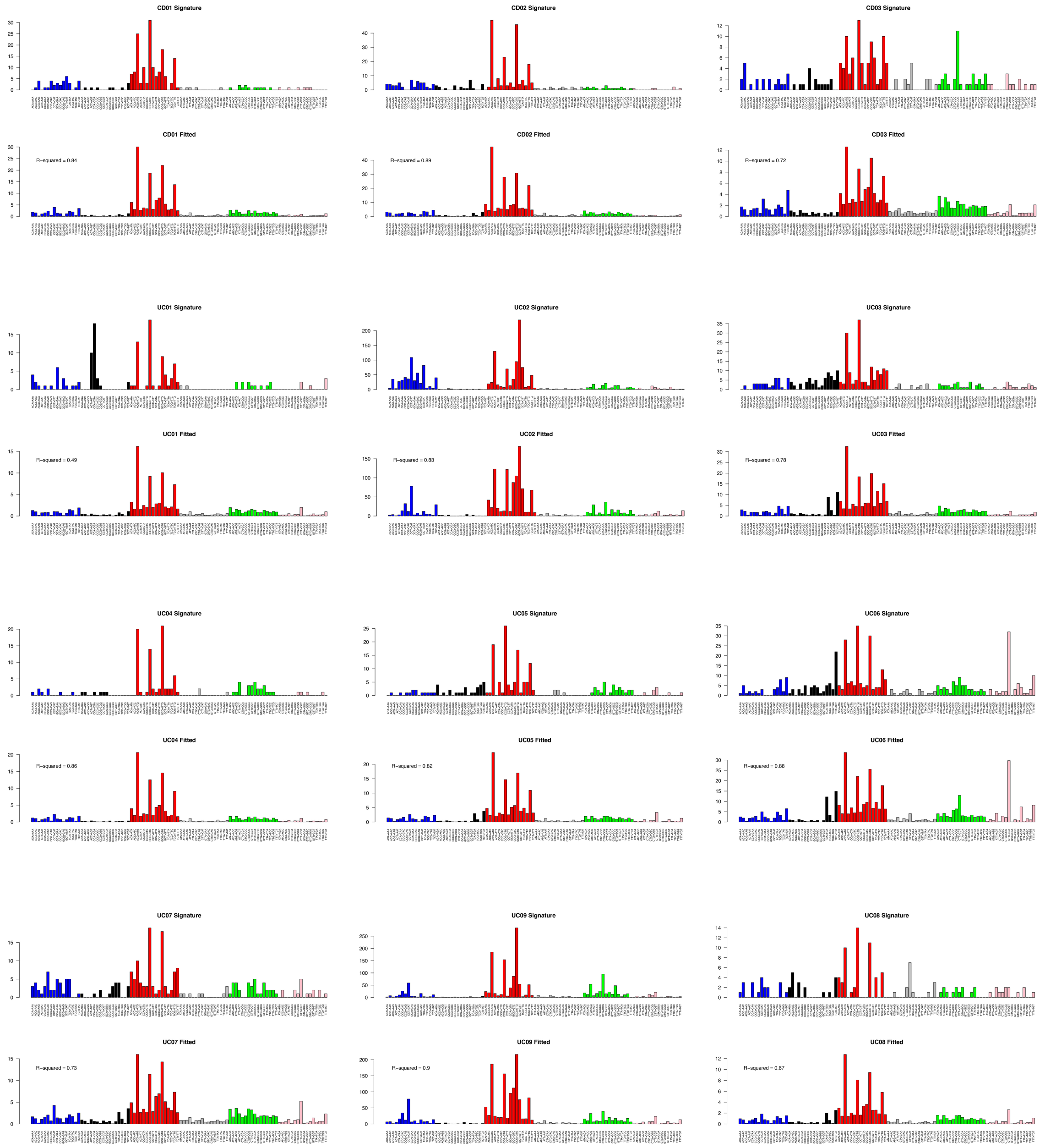

Supplementary Figure 6 – Composite mutational signature analyses of CA-CRCs

A. 96-channel classification of CA-CRCs defined by the substitution class and sequence context immediately 3′ and 5′ to the mutated base. Mutation types are on the horizontal axis and the vertical axis depicts the percentage of mutations attributed to a specific mutation type.

B. Stacked bar chart displaying the proportional contribution of each signature to the CA-CRCs.

C. and D. Comparison of absolute (**C.**) and relative (**D.**) contribution of each signature in pre-cancer SNAs, clonal ('early') SNAs and subclonal ('late') SNAs. None of the differences are significant by the Mann Whitney test (*p*>0.05).

E. and F. Comparison of absolute (**E.**) and relative (**F.**) contribution of each signature in MSS and MSI CA-CRCs vs. S-CRCs. None of the differences are significant by the Mann Whitney test (*p*>0.05).

Supplementary Figure 6

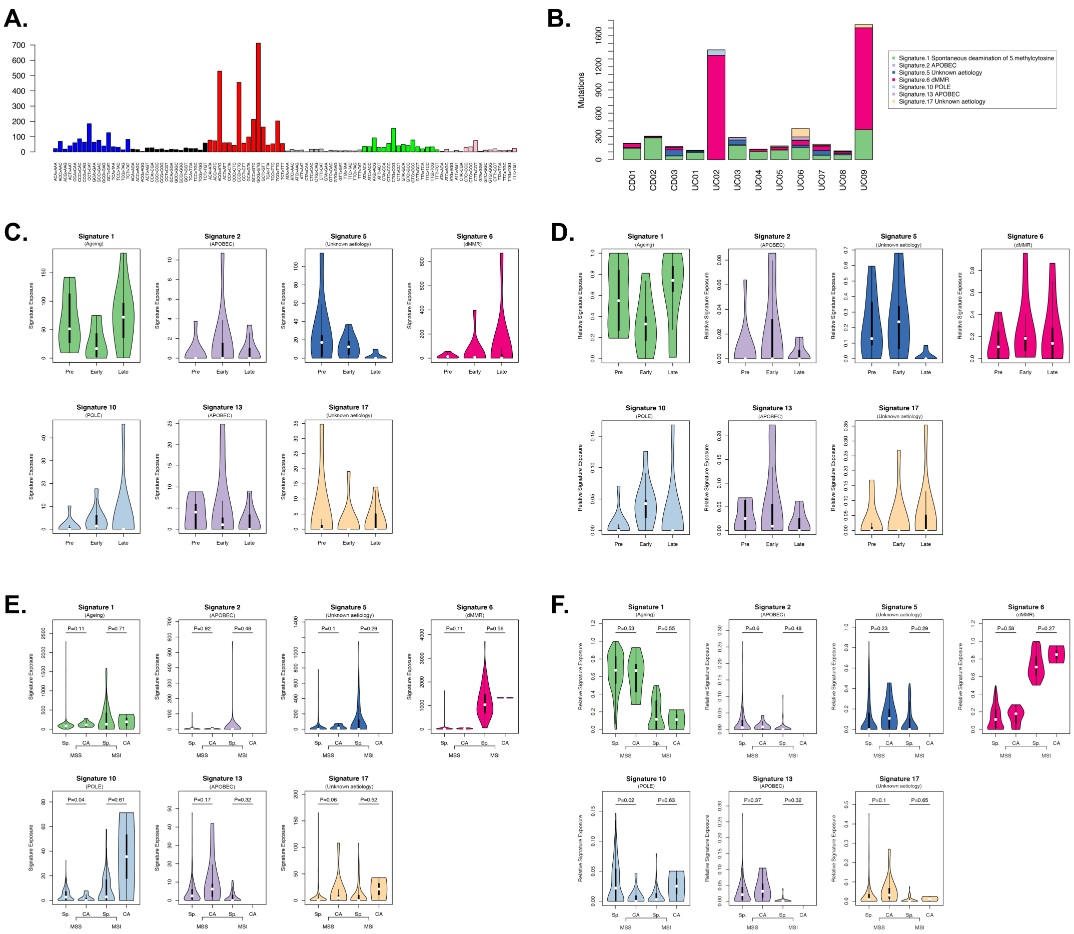

Supplementary Figure 7 – Copy number validation by FISH
Representative images of FISH performed on FFPE sections of carcinomas with copy number (CN) 2 (**A**), 3 (**B**) and 4 (**C**) at the loci of interest. White arrowheads indicate cells that were included in the analysis.

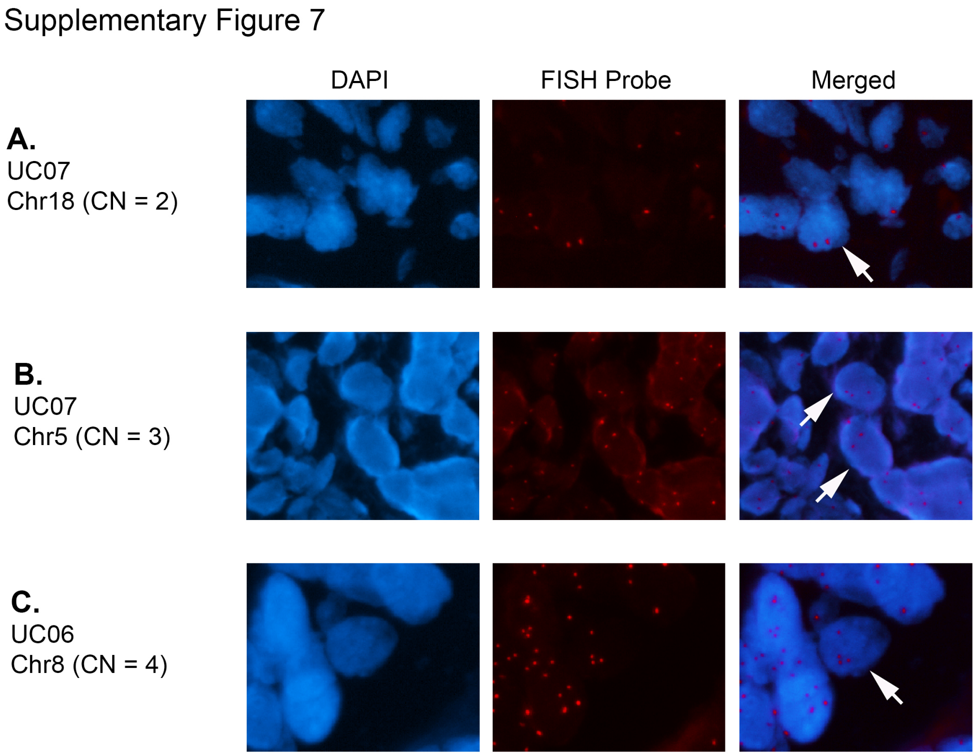

Supplement: Supplementary data [file gutjnl-2018-316191supp004.pdf]
